# Supplementary material for: Transplantation of genome-edited retinal organoids restores some fundamental physiological functions coordinated with severely degenerated host retinas
Source: Stem Cell Reports. 2025 Jan 16;20(2):102393. doi: 10.1016/j.stemcr.2024.102393 (PMC11864131; doi:10.1016/j.stemcr.2024.102393)
Supplement: Document S1. Figures S1–S4, Tables S1 and S2, and supplemental procedures [file mmc1.pdf]

Stem Cell Reports, Volume *20*

Supplemental Information

Transplantation of genome-edited retinal organoids restores some fundamental physiological functions coordinated with severely degenerated host retinas

Mikiya Watanabe, Takayuki Yamada, Chieko Koike, Masayo Takahashi, Masao Tachibana, and Michiko Mandai

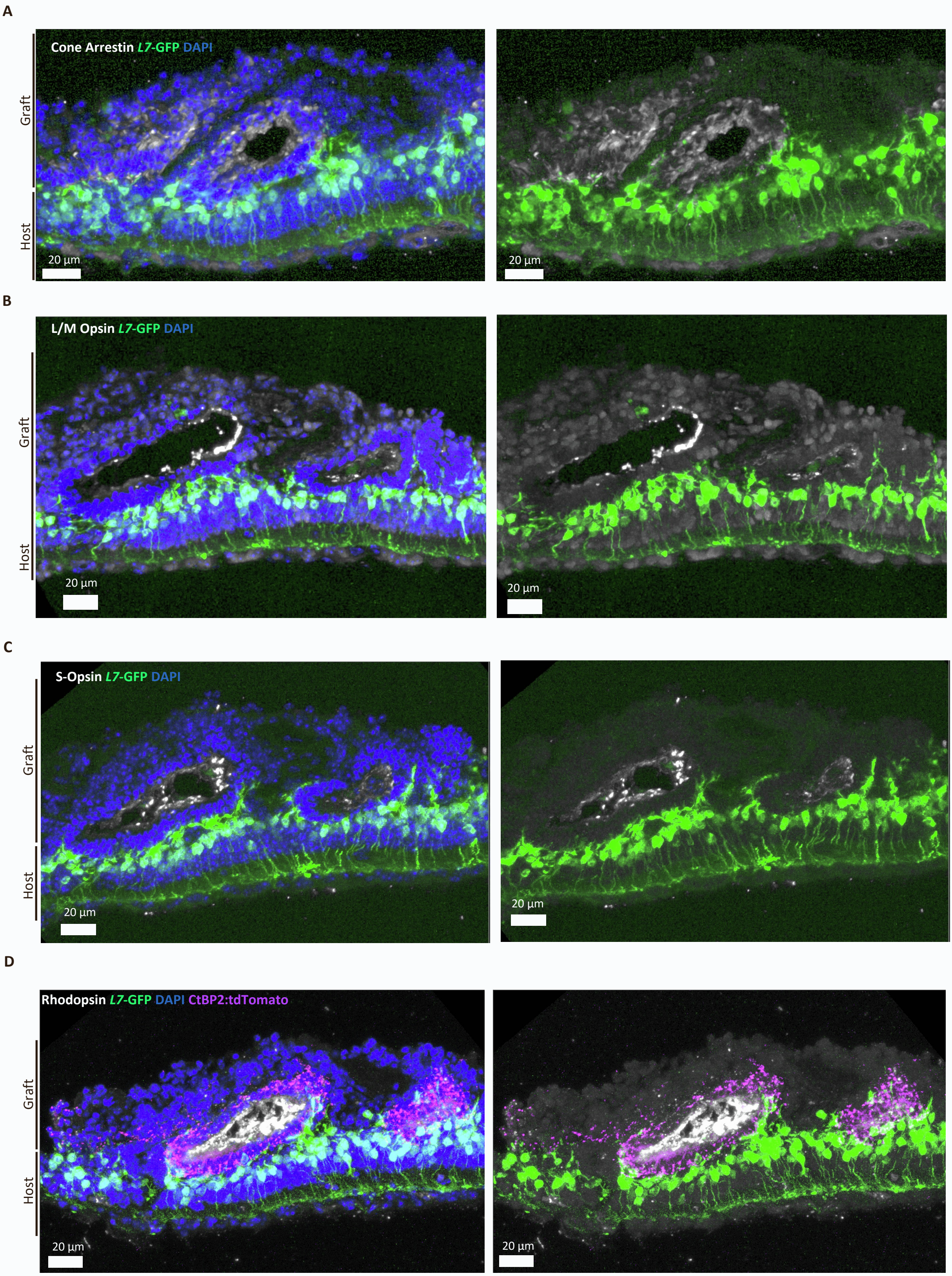

Figure S1. Immunostaining of TP-*rd1* retinal sections

(A-D) Immunostaining for TP-*rd1* retina with cone arrestin, L/M opsin, S-opsin and rhodopsin.

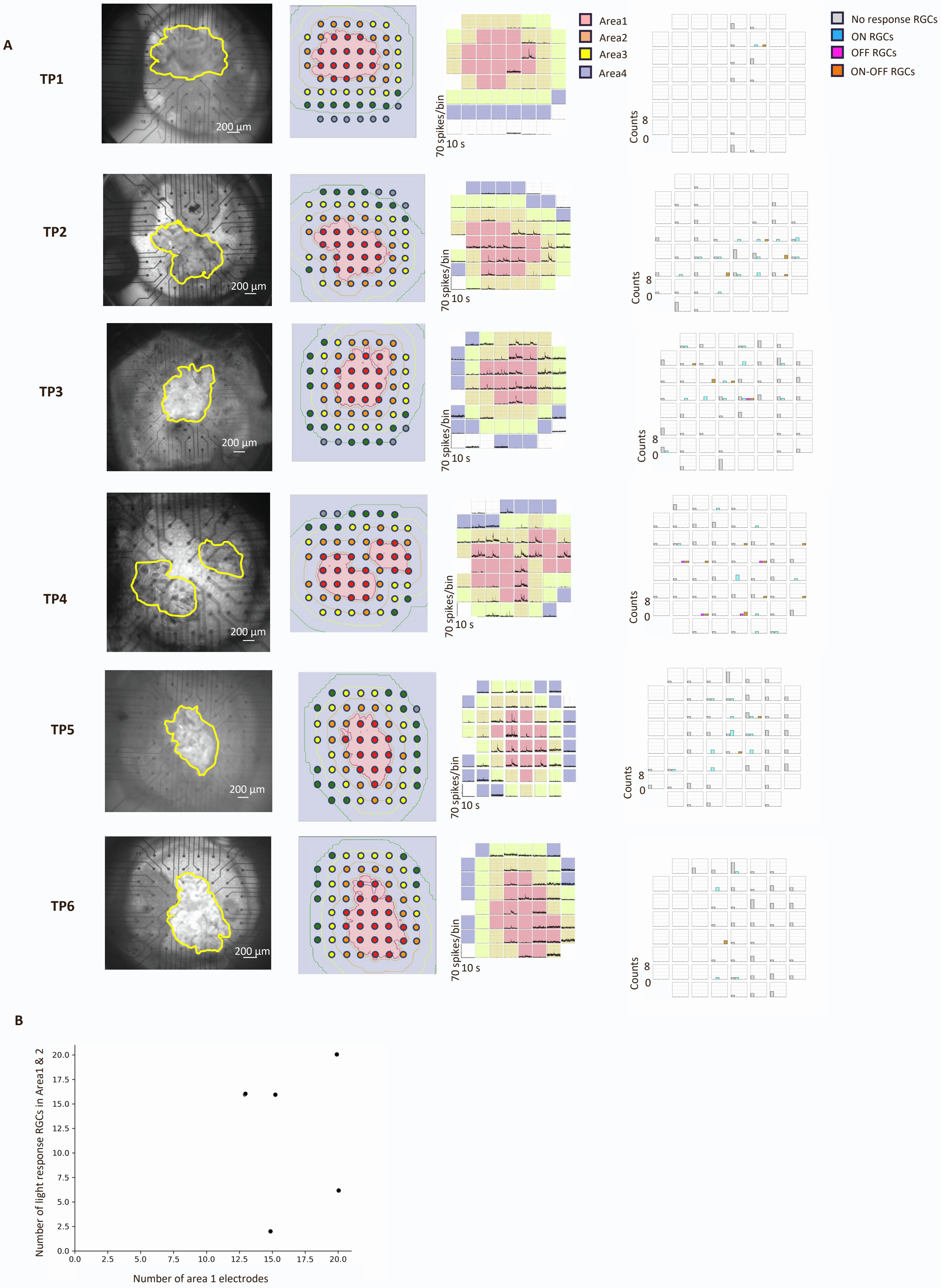

Figure S2. Summary of each TP-*rd1* retina recorded by MEA

(A)Photographs of the retina with CtBP2:tdTomato fluorescence (except for TP4 with Infra-Red image) during MEA recordings for each TP-*rd1*, along with mapping results and the time histogram of spikes

(B) Relationship between the number of light responses RGCs in area 1-2 and the number of electrodes in area 1.

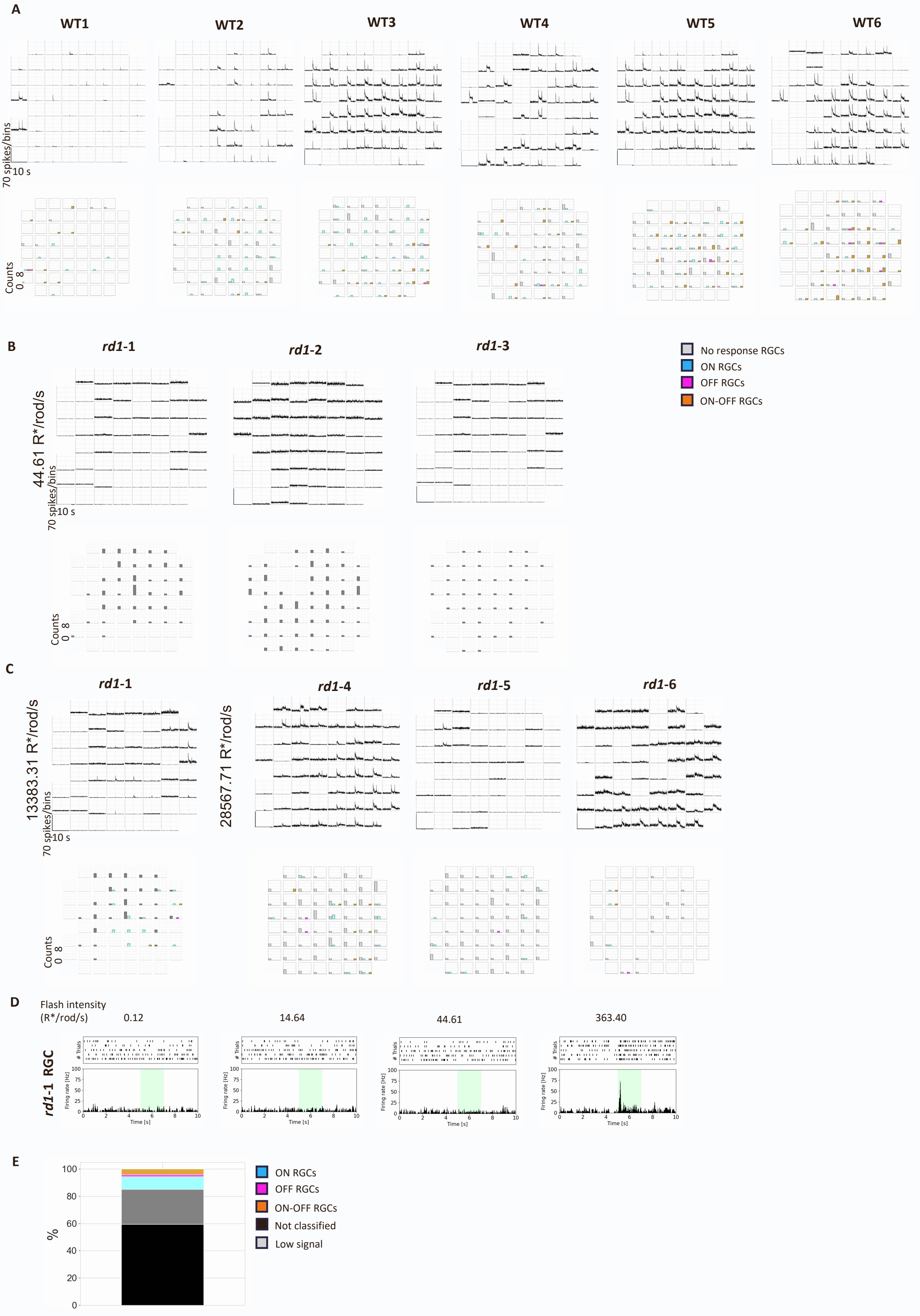

Figure S3. Summary of each WT and *rd1* retina recorded by MEA

(A-C) The time histogram of spikes recorded from each electrode and distribution map illustrating the counts of each RGC type after spike sorting in WT retinas (A:6 retinas, 6 animals), 4-6 weeks *rd1* retinas(B: 3 retinas ,3 animals, C: 4 retinas, 4 animals).

(D) Representative light responses of RGC in 6 week *rd1* retina.

(E) RGC types in 4-6 weeks *rd1* retinas (288 cells from 3 retinas, 3 animals).

Based on the shape of the response to a 2-s flash (28567.71 R*/rod/s), each RGC was classified as ON(27 cells), OFF (4 cells), ON-OFF(12 cells), Low-signal (74 cells), or Not classified (171 cells).

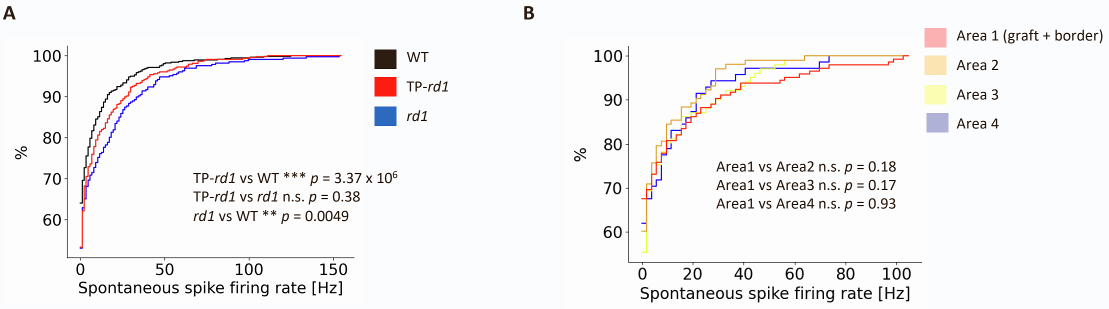

Figure S4. Spontaneous activity in the dark from WT, *rd1* and TP-*rd1* retinas

(A) Cumulative probability of 10-second mean spontaneous spike firing rate under dark from WT(556 RGCs, 6 retinas, 6 animals), the TP-*rd1* (413 RGCs, 6 retinas, 6 animals) and the *rd1* (326 RGCs, 4 retinas, 4 animals) retinas.

(B) Cumulative probability of 10-second mean spontaneous spike firing rate under dark from Area1 (147 RGCs), Area2 (102 RGCs), Area3 (94 RGCs) and Area4 (70 RGCs) in the TP-*rd1* retinas (6 retinas, 6animals).

Table S1. Summary of each TP-*rd1* retina used for the experiment

Sample	Host	Age at analysis (Weeks)	Age at time of transplantation (Weeks)	Post-TP (weeks)	graft Cell Line	Graft Age	No. of Electrodes				No.of light-responded RGCs / ALL RGCs after spike sorting			
							Area1	Area2	Area3	Area4	Area1	Area2	Area3	Area4
TP1	*rd1*	19	10	9	*Islet1* $^{-/-}$	dd13	15	15	14	8	0/8	2/5	0/1	0/1
TP2	*rd1*	25	19	6	*Islet1* $^{-/-}$	dd13	15	17	16	8	9/28	7/16	4/18	0/5
TP3	*rd1*	22	14	8	*Islet1* $^{-/-}$	dd13	13	17	13	12	12/28	4/27	1/15	2/24
TP4	*rd1*	22	14	8	*Islet1* $^{-/-}$	dd13	20	15	13	9	12/32	8/20	2/11	2/11
TP5	*rd1*	19	10	9	*Islet1* $^{-/-}$	dd13	13	13	17	15	12/23	4/17	1/28	0/23
TP6	*rd1*	18	10	8	*Islet1* $^{-/-}$	dd13	20	15	15	9	4/28	2/17	1/21	0/6
TP7*	*rd1*	46	32	14	*Islet1* $^{-/-}$	dd13	-	-	-	-	-	-	-	-

*TP7 was utilized solely for the histological analysis presented in Figure S1.

Table S2. Summary of Mann–Whitney U test in Figure3D

Flash intensity R*/rod/s	p-values	Number of RGCs	
		WT	TP-*rd1*
1.49	1.08×10^{-9}	175	25
4.32	1.76×10^{-8}	224	23
15.32	2.59×10^{-6}	251	62
49.38	0.0047	251	66
95.22	1.29×10^{-5}	279	84

Supplemental procedures

Mouse ES cell line and retinal organoid differentiation

On differentiation day (DD) 0, mouse ES cells were plated at 5,000 cells/well in 96-well plates (Thermo, 174925) to form aggregates in differentiation medium (Glasgow minimum essential medium [GIBCO, 11710035], 5 % KSR [GIBCO, 10828-028], 0.1 mM nonessential amino acids [GIBCO, 11140-050], 1 mM pyruvate [Sigma, S-8636], and 0.1 mM 2-mercaptoethanol [Wako,137-06862]) + 100 μM AGN193109 (Toronto Research Chemicals, A427000). The aggregates were incubated at 37 °C with 20 % O_2 and % CO_2. On DD1, growth factor-reduced Matrigel (2 %, BD Biosciences, 354230) was added to the differentiation medium. On DD7, aggregates were transferred to the retinal maturation medium (DMEM/F12 with GlutaMAX [GIBCO, 10565], 1 % N2 supplement [GIBCO, 17502-048] and 1 % penicillin-streptomycin [GIBCO, 15140-122]) in a 6-cm dish (CORNING, 351007) and incubated at 37 °C with 40 % O_2 and 5 % CO_2. On DD11, all-trans retinoic acid (0.5 μM, Sigma, R2625-100MG) and L-taurine (1 mM, Sigma, T8691) were added to the retinal maturation medium. On DD13, a small piece (approximately 1 × 0.5 mm) containing a characteristic continuous neural epithelial structure was excised from each optic vesicle for transplantation (Assawachananont et al. 2014).

Transplantation of gRO sheets

Injection tips were prepared from disposable micropipettes (Drummond, 1-000-0500) using a micropipette puller (Sutter Instrument, P-97/IVF Puller), and the tip (approximately 500 μm in diameter) was sharpened using a microgrinder (Narishige, EG-400). The injection tip was then fixed in a microelectrode holder (World Precision Instruments, MPH310) on the electrode handle (World Precision Instruments, 2505), which was connected to an extension tube, and the route was filled with HBSS (Gibco, 14170112) using a 1-mL syringe. Then, the syringe was replaced

by a 10-µl micro-syringe (Hamilton, 1701LT), and the gRO sheets were loaded in the injection tip by aspiration with approximately 2 µL 6x Viscoat, hyaluronate and chondroitin sulfate sodium (Alcon). Mice were anesthetized with a mixture of medetomidine hydrochloride (0.75 mg/kg body weight; Nippon Zenyaku Kogyo Co., Ltd., Domitor), midazolam (4 mg/kg body weight; Maruishi Pharmaceutical, Dormicum) and butorphanol tartrate (5 mg/kg body weight; Meiji Animal Health, Vetorphal). The eyes were dilated with 0.4 % tropicamide (Rohto Nitten). Two small scleral punctures were made at the peripheral retina with a 30 G needle, one for graft injection and the other for reducing intraocular pressure, and the gRO sheets were gently injected subretinally into the eyes of *rd1* or *L7*-GFP:*rd1* mice.

Immunohistochemistry

After MEA recordings, the retina was removed from the MEA, washed with phosphate buffered saline (PBS) and fixed with 4% paraformaldehyde (Wako Pure Chemical Industries, 30525-89-4) for 15 min at room temperature (RT). The retina was then incubated in blocking buffer (3 % Triton X-100 and 1 % bovine serum albumin [Sigma, A4503]) for 1 h at RT, and then with the primary antibody against mGluR6 (1:2000) (Leinonen et al., 2020) for 1 week at 4 °C. The retina was washed three times with blocking buffer, followed by incubation with secondary antibody (Alexa Fluor 647 Donkey Anti-Sheep IgG (H+L), Invitrogen, A21448) and DAPI (Invitrogen, D1306) for 3 days at 4 °C.

For immunostaining of the TP-*rd1* retinal sections, mice were sacrificed by cervical dislocation and the eyes were enucleated. The lenses were excised, and the eyes were immediately fixed in 4 % paraformaldehyde for 15 min. The eyes were then washed with PBS and immersed in a 30 % sucrose solution overnight at 4 °C. Subsequently, they were embedded in optimal cutting temperature compound (4583, Sakura Finetek, Tokyo, Japan) and stored at -30 °C. Coronal

cryosections of 14 μm thickness were prepared using a cryostat (Thermo Scienctific CryoStar NX70). The sections were then incubated in blocking buffer (3 % Triton X-100 and 1 % bovine serum albumin [Sigma, A4503]) for 1 h at room temperature (RT), followed by incubation with primary antibodies against cone arrestin (Millipore, AB15282), rhodopsin (sigma, 04886), L/M opsin(Opsin Red/Green, Millipore, AB5405), and S-opsin(OPN1SW, SantaCruz, sc-14363) for 3 days at 4 °C. The retina was washed three times with the blocking buffer, followed by incubation with secondary antibody donkey-anti-Rb-647 (Invitrogen, A31573), donkey-anti-mouse-647 (Invitrogen, A31571), donkey-anti-Goat-647 (Invitrogen, A21447), and DAPI (Invitrogen, D1306) for 1 day at 4 °C.

After the secondary antibody incubation, the retina was washed three times with blocking buffer and subsequently mounted on a glass slide using Vectashield (Vector Laboratories, H-1000). Z-stack Images were acquired with a Leica TCS SP8 confocal microscope and reconstructed in 3D using Imaris Microscopy Image Analysis Software (Oxford Instruments, http://www.bitplane.com/). An overall view of the engrafted whole-mount retina was obtained using a BZ9000 (Keyence).

Spike sorting

The raw data were band-pass filtered to extract components between 100 and 3,000 Hz. The maximum negative value below the threshold defined by the following formula was detected as the spike peak (Quiroga et.al., 2004).

$$threshold = -4 \times median$$

where *x* represents each data point. The first and second differences between data points were calculated from data points between −1 and 2 ms from the peak of the detected spike, and the dimensions were converted to two dimensions using principal component analysis. Clustering

was performed using density-based spatial clustering of applications with noise as a feature (HDBSCAN) of each waveform. Furthermore, using a template matching method (Zhang et al., 2004), we implemented an algorithm to merge clusters and another to rescue spikes from a group of waveforms considered as noise into one of the clusters. To verify the accuracy of spike sorting, we performed autocorrelation analysis on the sorted spike train from each unit and confirmed the presence of a refractory period (± 1 ms).

Light simulation for MEA recordings

In Figure 2, a 2-s flash (95.22 R*/rod/s) was presented under the dark background. In Figure 3, to examine the light intensity-response relationship, the 2-s flash intensity was varied from 0.83 to 95.22 R*/rod/s. In a similar experiment, the *rd1* retina (4–6-week-old) was stimulated by light (24.85 to 28567.71 R*/rod/s). In Figures 4–6, the background light was applied for more than 5 min before the start of the measurements. In Figure 4, a 2-s flash (18794.68 to 43944.81 R*/rod/s) was applied under the photopic light background (15441.33 R*/rod/s). In Figure 5 and 6, a 10-ms square wave stimulus was flickered at 5, 15, 20, and 25 Hz. The light intensities used were: 15.32 R*/rod/s flicker stimulus under the dark background condition, 95.23 R*/rod/s flicker stimulus under the dark background condition, and 43944.81 R*/rod/s flicker stimulus under the 1189.59 R*/rod/s background light condition. In the actual experiment, after completing the stimulation protocol under the dark background, the samples were given a 5-min adaptation period to the light background, followed by the administration of the stimulation protocol under the light background. A 2-s flash (0.12 to 2856.71 R*/rod/s) under the dark background is shown in Figure S3.

Supplemental references

Assawachananont, J., Mandai, M., Okamoto, S., Yamada, C., Eiraku, M., Yonemura, S., Sasai, Y., and Takahashi, M. (2014). Transplantation of embryonic and induced pluripotent stem cell derived 3D retinal sheets into retinal degenerative mice. Stem Cell Reports. *2*, 662–674._ https://doi.org/10.1016/j.stemcr.2014.03.011.

Leinonen, H., Pham, N. C., Boyd, T., Santoso, J., Palczewski, K., & Vinberg, F. (2020). Homeostatic plasticity in the retina is associated with maintenance of night vision during retinal degenerative disease. Elife, 9, e59422. https://doi.org/10.7554/eLife.59422

Quiroga, R. Q., Nadasdy, Z., and Ben-Shaul, Y. (2004). Unsupervised spike detection and sorting with wavelets and superparamagnetic clustering. Neural Comput, 16, 1661-–1687. https://doi.org/10.1162/089976604774201631.

Zhang, P. M., Wu, J.- Y., Zhou, Y., Liang, P.- J., and Yuan, J.- Q. (2004). Spike sorting based on automatic template reconstruction with a partial solution to the overlapping problem. J Neurosci Methods, 135, 55-–65. https://doi.org/10.1016/j.jneumeth.2003.12.001.
