# Supplementary material for: Transplantation of genome-edited retinal organoids restores some fundamental physiological functions coordinated with severely degenerated host retinas
Source: Stem Cell Reports. 2025 Jan 16;20(2):102393. doi: 10.1016/j.stemcr.2024.102393 (PMC11864131; doi:10.1016/j.stemcr.2024.102393)
Supplement: Document S2. Article plus supplemental information [file mmc2.pdf]

Stem Cell Reports
Article
OPEN ACCESS

Transplantation of genome-edited retinal organoids restores some fundamental physiological functions coordinated with severely degenerated host retinas

Mikiya Watanabe,[1,2,3] Takayuki Yamada,[3,4] Chieko Koike,[5,6,7] Masayo Takahashi,[4,8] Masao Tachibana,[5] and Michiko Mandai[9,10,11,12,*]
[1]VCCT Inc., Kobe, Hyogo 650-0047, Japan
[2]Graduate School of Pharmacy, Ritsumeikan University, Kusatsu, Siga 525-8577, Japan
[3]Cell and Gene Therapy in Ophthalmology Laboratory, BZP, RIKEN, Wako, Saitama 351-0198, Japan
[4]Vision Care Inc., Kobe, Hyogo 650-0047, Japan
[5]Center for Systems Vision Science, Organization of Science and Technology, Ritsumeikan University, Kusatsu, Shiga 525-8577, Japan
[6]Ritsumeikan Global Innovation Research Organization(R-GIRO), Ritsumeikan University, Kusatsu, Siga 525-8577, Japan
[7]College of Pharmaceutical Science, Ritsumeikan University, Kusatsu, Siga 525-8577, Japan
[8]Ritsumeikan Advanced Research Academy, Ritsumeikan University, Kusatsu, Shiga 525-8577, Japan
[9]Research Center, Kobe City Eye Hospital, Kobe, Hyogo 650-0047, Japan
[10]Research Organization of Science and Technology, Ritsumeikan University, Kusatsu, Shiga 525-8577, Japan
[11]Laboratory for Animal Resources and Genetic Engineering, RIKEN Center for Biosystems Dynamics Research, Kobe, Hyogo 650-0047, Japan
[12]Lead contact
*Correspondence: e_lab.mandai@kcho.jp
https://doi.org/10.1016/j.stemcr.2024.102393

SUMMARY

We have previously shown that the transplantation of stem cell-derived retinal organoid (RO) sheets into animal models of end-stage retinal degeneration can lead to host-graft synaptic connectivity and restoration of vision, which was further improved using genome-edited *Islet1*$^{-/-}$ ROs (gROs) with a reduced number of ON-bipolar cells. However, the details of visual function restoration using this regenerative therapeutic approach have not yet been characterized. Here, we evaluated the electrophysiological properties of end-stage *rd1* retinas after transplantation (TP-*rd1*) and compared them with those of wild-type (WT) retinas using multi-electrode arrays. Notably, retinal ganglion cells (RGCs) in TP-*rd1* retinas acquired light sensitivity comparable to that of WT retinas. Furthermore, RGCs in TP-*rd1* retinas showed light adaptation to a photopic background and responded to flickering stimuli. These results demonstrate that transplantation of gRO sheets may restore some fundamental physiological functions, possibly coordinating with the remaining functions in retinas with end-stage degeneration.

INTRODUCTION

Retinitis pigmentosa is a group of inherited diseases in which photoreceptors are progressively lost, and nearly a hundred genes have been reported as causal genes so far (https://web.sph.uth.edu/RetNet). Typically, rod photoreceptors primarily degenerate starting from the mid-peripheral retina, followed by the loss of cone photoreceptors in the central macula, which can lead to complete blindness. Recently, several treatment strategies have been developed including gene therapies, optogenetics, retinal prosthetics, and stem cell therapies. Gene therapies generally aim to restore the function of photoreceptor cells by providing the missing proteins or correcting the genetic mutations present before significant cell loss occurs (Botto et al., 2022). In contrast, optogenetic approaches target advanced degeneration and use the ectopic expression of light-signaling proteins, including microbial opsins and channelrhodopsins, in surviving retinal cells other than photoreceptors (Bi et al., 2006; Lagali et al., 2008; Sahel et al., 2021). The retinal prosthesis captures images using a camera and conveys visual information via the electrical stimulation of the remaining retinal ganglion cells (RGCs) (Ayton et al., 2020). Optogenetics and artificial retinas are robust methods that can replace physiological retinal functions. However, these approaches drive RGCs or bipolar cells (BCs) regardless of the cell subtype (e.g., they do not distinguish between ON and OFF cells), which may result in unnatural activation patterns and may not reproduce innate retinal information processing. Stem cell therapies aim to replace lost/degenerated cells by transplanting pluripotent stem cell-derived photoreceptors that use the remaining retinal circuitry and hopefully restore visual processing, at least partially (Mandai, 2023).

We previously reported that visual function could be restored by transplanting mouse retinal organoid (RO) sheets into mouse models of end-stage retinal degeneration (*rd1*) whose visual function had already been lost (Mandai et al., 2017). Using the rod BC reporter mouse line (*L7*-GFP) and the synapse reporter mouse embryonic stem (ES)/induced pluripotent stem (iPS) cell line (*Nrl*-CtBP2:tdTomato), we found that synaptic connectivity between the host BCs and grafted photoreceptors was partially established. Multi-electrode array (MEA) recordings revealed

Published by Elsevier Inc. on behalf of International Society for Stem Cell Research.
This is an open access article under the CC BY license (http://creativecommons.org/licenses/by/4.0/).

that host RGCs underneath the grafted area responded to light stimulation. Furthermore, the transplanted mice exhibited light-responsive behavior. Recently, we also observed the microstructures of these host-graft synaptic contact sites (*L7*-GFP and *Nrl*-CtBP2:tdTomato) by electron microscopy using a correlative array tomography technique, and we were able to confirm the invagination of host BC dendrites toward mature graft ribbons, potentially accompanying the presence of synaptic complex structures and preceding horizontal cell process invagination (Akiba et al., 2024). Based on similar observations using immunohistochemistry in the xenotransplantation of human ES/iPS cell (iPSC) RO sheets in nude rat retinal degeneration models (Watari et al., 2023), a clinical study was conducted to test the safety of iPSC-derived RO sheets for retinitis pigmentosa at Kobe Eye Center Hospital in 2020. The clinical study showed the safety and stable survival of grafted sheets over 2 years in two cases of advanced retinitis pigmentosa (Hirami et al., 2023).

RO sheets used in the previous clinical study develop retinal inner cells, including BCs, horizontal cells, and Müller cells, after transplantation. These inner cells appear to contribute to the maturation of photoreceptors in the grafted sheets. However, BCs in the grafted sheets seem to competitively impede synapse formation between BCs in the host retina and photoreceptors in the grafted sheets. To solve this problem, we made genome-edited ROs (gROs), in which the number of rod BCs or ON BCs was reduced after transplantation by deleting *bhlhb4* or *Islet1*, respectively (Matsuyama et al., 2021; Yamasaki et al., 2022). In mice transplanted with gRO sheets, the number of photoreceptor synapses per host BC increased, and spontaneous RGC activities were reduced compared to those in mice transplanted with non-genome-edited RO (non-gRO) sheets. The *rd1* mice with gRO grafts showed better visual performance than mice with non-gRO grafts. These observations suggest that gRO grafts may achieve simple transmission of light signals to RGCs and coordinately interact with the surrounding neural network in the host retina.

An important feature of the visual system is its ability to process light signals at various spatiotemporal frequencies over a wide range of background light intensities. The degeneration of photoreceptors causes remodeling of neural circuits in the retina (Jones et al., 2016; Kalloniatis et al., 2016). However, the extent to which basic retinal function can be recovered using a regenerative therapeutic approach has not yet been determined.

Here, we transplanted mouse gRO sheets into end-stage retinal degeneration mice, *rd1*, and by using MEAs to the isolated retinal preparation, we examined in detail the extent to which visual functions were restored. We found that RGCs in *rd1* retinas transplanted with gRO sheets (TP-*rd1*) induced ON, OFF, and ON-OFF responses to light stimulation. TP-*rd1* retinas also showed light intensity-dependent function by adapting to scotopic-mesopic and photopic background lights. Furthermore, recordings of micro-electroretinograms (mERGs) and RGC firing in TP-*rd1* retinas revealed that responses could follow flickering light stimuli. These results demonstrate that transplantation of gRO sheets is useful for restoring some fundamental physiological functions in the retina with end-stage degeneration.

RESULTS

Transplantation of gRO sheets restores RGC responses to flashing light in the *rd1* mice

rd1 mice are progressive retinal degeneration models that lose most photoreceptors in the first 4 weeks after birth (Fujii et al., 2016; Matsuyama et al., 2021). gRO sheets were prepared from the *Islet1*$^{-/-}$ *Nrl*-CtBP2:tdTomato reporter mouse ES line (Matsuyama et al., 2021) and transplanted into C57BL/6J-Pde6b^{rd1-2J} mice (referred to as *rd1*) aged $\geq$10 weeks. Using the retina obtained from the mouse $\geq$6 weeks after transplantation, we examined whether the transplanted gRO sheets made synaptic connections with the *rd1* retina by performing whole-mount staining of the transplanted retina after MEA recordings. Similar to our previous reports (Mandai et al., 2017; Matsuyama et al., 2021), we found that although the transplanted gRO sheets formed varying degrees of rosette-like structures, the transplanted gRO sheets made synaptic connections with host BC dendrites (visualized by expression of *L7*-GFP and the postsynaptic marker mGluR6 at the tip of the dendrites) in all observed samples (n = 4 retinas, Figures 1A–1C). Photoreceptor cells in the graft were positive for S-opsin, L/M-opsin, cone arrestin, and rhodopsin. Opsins were characteristically observed in the outer segment-like structures inside the rosettes, similar to our previous observations (Figures S1A–S1D).

To investigate the physiological functions of the TP-*rd1* retina, a 2-s flash stimulus was applied to the isolated retina under the dark background condition, and RGC activity was recorded using the MEA system (Figures 2 and S2). The grafted area on the MEA was identified by CtBP2:tdTomato fluorescence expressed at the synaptic terminals of photoreceptors in the graft (area 1; Figures 2A, 2B, and S2). The areas surrounding the graft were subdivided into areas 2, 3, and 4 (Figures 2B and S2, see Experimental procedures). Light-evoked firing was recorded from each electrode in both wild-type (WT) and transplanted retinas (Figure 2B, TP-*rd1* retina; Figure 2C, WT retina). In TP-*rd1* retinas, light-evoked firing was most frequently observed in area 1.

Light-evoked responses were classified into “ON,” “OFF,” “ON-OFF,” “low signal,” and “not classified” types after

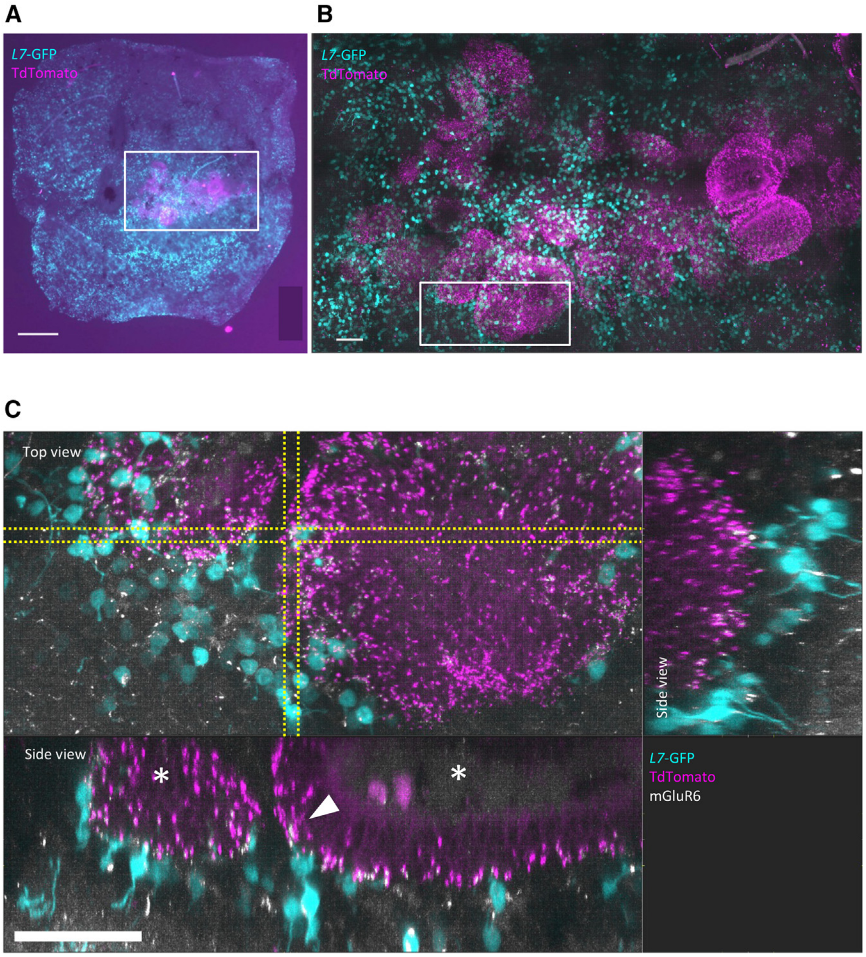

Figure 1. *Islet1$^{-/-}$* genome-edited retinal organoid sheets transplanted into the *rd1* retina suggest synaptic reconstruction

(A) Overall image of the genome-edited retinal organoid (gRO)-engrafted *rd1* retina after MEA recording. *L7*-GFP-positive host bipolar cells (cyan) are distributed throughout the host retina, whereas presynaptic CtBP2:tdTomato expressed (magenta) under the *Nrl* promoter shows the presence of engrafted mature photoreceptors. A whole-mount view photographed using a fluorescence microscope (BZ9000). White box indicates the grafted area. Scale bar, 300 μm.

(B) Magnified view of the white square in (A). A segmented image of the host-graft interface area processed from the z stack image using confocal microscopy (Leica TCS SP8). Scale bar, 50 μm.

(C) Magnified view of the white square in (B). *L7*-GFP-positive host bipolar cell dendrites expressing the mGluR6 (white) contact (arrowheads) presynaptic marker *Nrl*-CtBP2:tdTomato (magenta) expressed at the synaptic ribbons in the photoreceptor axon terminals in the graft. Bottom and top right images are the side views of the section between 2 lines in "*En face* view" image on the top left. The transplanted gRO sheets formed varying degrees of rosette-like structures (asterisks). Scale bar, 50 μm.

spike sorting (Figures 2D and 2E, see Experimental procedures). However, the proportion of ON-OFF type response was smaller in TP-*rd1* retinas than that in WT retinas (Figure 2E). RGC responses were occasionally detected outside the grafted areas i.e., areas 2–4 (Figure 2E). RGCs outside the grafted area might have received input from BCs in the border area or from outside the grafted area, as suggested in our previous studies (Mandai et al., 2017; Matsuyama et al., 2021). Additionally, the grafted area may potentially correlate with RGC responses, but currently, we only have a limited number of samples in this study, with two retinas showing an overall low response possibly due to some technical problems of the MEA recording, which was occasionally observed in the WT retinas as well (Figure S3).

Degenerated retinas are characterized by increased spontaneous activity owing to a lack of photoreceptor inputs (Biswas et al., 2014; Trenholm and Awataramani, 2015; Tu et al., 2015). Consistent with previous reports, the spontaneous firing rate of RGCs in *rd1* mice was found to be significantly higher than that in WT mice (Figure S4A). Similarly, the spontaneous firing rate in TP-*rd1* mice was also markedly higher than that observed in WT mice (Figure S4A). Conversely, no significant difference was observed between the TP-*rd1* and *rd1* mice. Additionally, no significant differences were observed among the different regions of TP-*rd1* retinal transplants in the current study (Figure S4B).

RGCs of TP-*rd1* mice respond to light stimulation in the scotopic-mesopic range

ON responses to different light intensities were recorded from ON and ON-OFF RGCs of WT, TP-*rd1* (recorded from areas 1–4), and *rd1* retinas (Figures 3A, 3B, S2, and S3). RGCs of TP-*rd1* retinas responded similarly to those of WT retinas. In contrast, RGCs of *rd1* retinas aged 4–6 weeks responded only to very strong light (Figures 3B and S3). In *rd1* mice, most rod photoreceptors degenerate within 1 month, whereas cone photoreceptors degenerate later (Sancho-Pelluz et al., 2008). Thus, the responses in

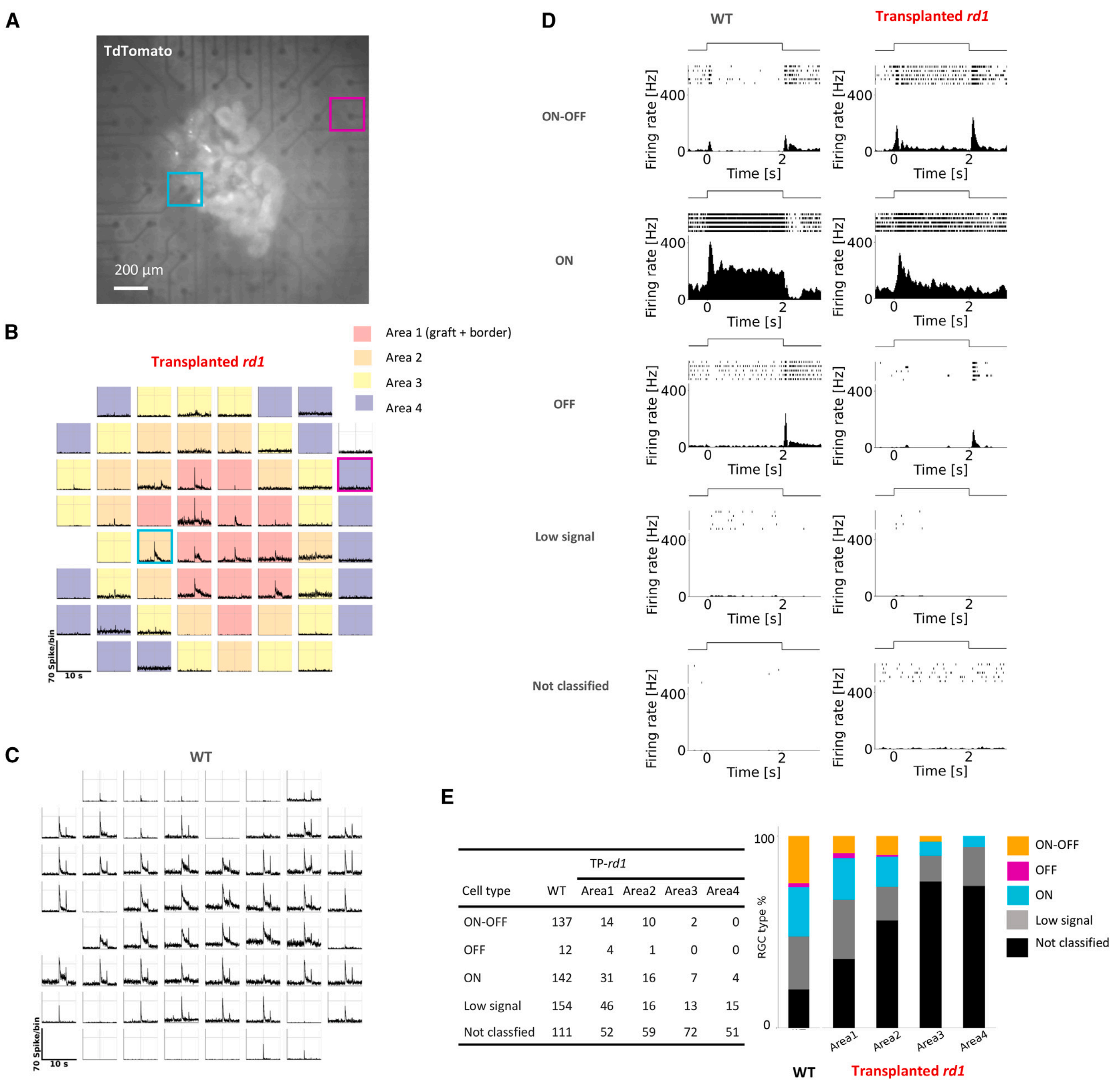

Cell type	WT	TP-*rd1* Area1	Area2	Area3	Area4
ON-OFF	137	14	10	2	0
OFF	12	4	1	0	0
ON	142	31	16	7	4
Low signal	154	46	16	13	15
Not classfied	111	52	59	72	51

Figure 2. ON, OFF, and ON-OFF RGC types were all observed in the transplanted *rd1* retina

(A)The transplanted *rd1* (TP-*rd1*) retina was placed RGC side down onto the MEA, and the grafted area was identified by the presence of CtBP2:tdTomato fluorescence.

(B and C) Time histogram of the spikes obtained from each electrode. (B) TP-*rd1* retina and (C) WT retina. Two-second flash stimulation was given between 5 and 7 s. We divided MEA areas into area 1 (graft and border), and areas 2–4 as described in Experimental procedures. The two square frames correspond to the positions shown in (A).

(D) Classification of RGC response types into ON, OFF, ON-OFF, low signal, and not classified in WT and transplanted *rd1* (TP-*rd1*) retinas after spike sorting. The low signal exceeded the threshold; however, the peak is < 10 Hz. RGCs not identified as any type were labeled as not classified. Each panel illustrates light stimulation, raster plots, and PSTHs, which were smoothed by a binomial filter for $n = 4$.

(E) RGC types in WT retinas (556 cells from 6 retinas, 6 animals), and those in area 1 (147 cells), area 2 (102 cells), area 3 (94 cells), and area 4 (70 cells) from the TP-*rd1* retinas (6 retinas, 6 animals).

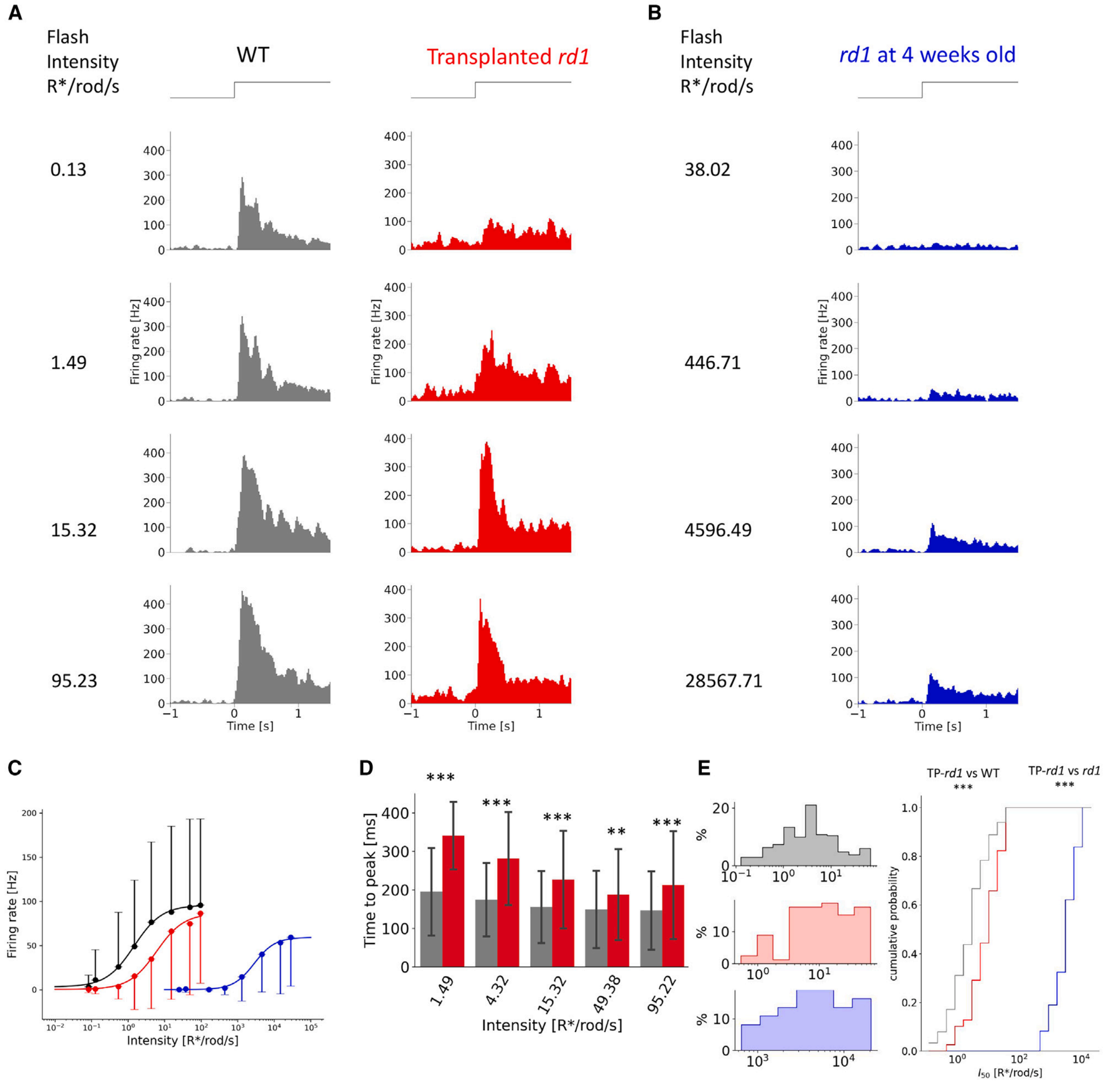

Figure 3. Light-evoked response properties of RGCs in the TP-*rd1* and WT retinas

(A and B) Representative RGC responses to flashes with different light intensities in WT, TP-*rd1* (22-week-old) (A), and 4-week-old *rd1* (B) retinas. PSTHs were smoothed by a binomial filter for $n = 4$.

(C) Relationship between mean firing rate of ON peak responses and light intensity in WT (black, 279 cells from 6 retinas, 6 animals), 18- to 25-week-old TP-*rd1* (red, 84 cells from 6 retinas, 6 animals), and 4- to 6-week-old *rd1* (blue 39 cells from 3 retinas, 3 animals) retinas. Error bars denote standard deviation.

(D) Time-to-peak latency of ON responses to stimuli with various light intensities. WT (gray) and TP-*rd1* (red) retinas (statistical values are listed in Table S2). Error bars denote standard deviation.

(E) Histogram and cumulative probability of intensity at half-maximum response (I_{50}) obtained from ON and ON-OFF RGCs in WT (268 RGCs, 6 retinas), TP-*rd1* (79 RGCs, 6 retinas, 6 animals), and *rd1* (39 RGCs, 3 retinas, 6 animals) retinas (*rd1* vs. TP-*rd1*: $p = 7.26 \times 10^{-31}$, WT vs. TP-*rd1*: $p = 2.81 \times 10^{-9}$, Kolmogorov-Smirnov test).

rd1 retinas at 4–6 weeks of age likely originate from the remaining cone photoreceptors.

We calculated the peak firing rate and time-to-peak latency of the peristimulus time histograms (PSTHs) obtained by stimulation with various light intensities (Figures 3C and 3D). In TP-*rd1* retinas, ON responses showed an increase in peak firing rate and shorter time-to-peak latency as the light intensity was increased in the scotopic-mesopic range, similar to that in WT retinas (Figures 3C and 3D). These responses were not observed in young *rd1* retinas, indicating that scotopic-mesopic light responses are likely to be newly acquired in TP-*rd1* retinas. The time-to-peak latency of ON responses in TP-*rd1* retinas at each light intensity was significantly longer than that in WT retinas, suggesting that *de novo* synapses may not be fully mature and/or have low efficiency.

The half-saturating intensity of ON responses was significantly lower by approximately 3 log units in TP-*rd1* retinas than in *rd1* retinas at 4–6 weeks of age, although half-saturating intensity was significantly higher by approximately 1 log unit in TP-*rd1* retinas than in WT retinas (Figure 3E). These results indicated that TP-*rd1* retinas may have acquired similar features as WT retinas in response to scotopic-mesopic light stimulation when compared to *rd1* retinas.

RGCs of the TP-*rd1* retina respond to light increment under the photopic background condition

In WT retinas, light adaptation causes a shift in the light intensity-response curve toward higher intensities. Next, we examined whether light-adapted TP-*rd1* retinas can still respond to light. We recorded ON responses to various light intensities from ON and ON-OFF RGCs of WT and TP-*rd1* retinas under the photopic background condition (15,441.33 R*/rod/s, Figure 4A). The peak firing rate of ON responses in TP-*rd1* retinas increased as the light intensity increased (Figure 4B). However, the time-to-peak latency was significantly longer than that in WT retinas (Figure 4C). Collectively, the TP-*rd1* retina could process light information not only under the dark background condition but also under the photopic background condition. The background light was strong enough to saturate the rod photoreceptors; thus, the observed responses were likely derived from the cones. Cells positive for cone-specific markers (cone arrestin, S-opsin, and L/M-opsin) were present within the photoreceptor rosettes, suggesting that the grafts contained functional cones capable of responding to flash stimuli even under a bright background light (Figures S1A–S1C).

Temporal resolution of the TP-*rd1* retina was partially recovered

To estimate the temporal resolution of light responses in TP-*rd1* retinas, we recorded local field potentials (mERGs) induced by flicker stimulation. Flicker stimulation induced periodic mERGs in WT retina (Figure 5A) and in (and near) the grafted area of TP-*rd1* retina (Figure 5B). We analyzed the extent to which the periodic mERGs followed the flicker stimulation by calculating the power spectrum (Figures 5C–5F). The peak frequency of the power spectrum corresponded to the stimulus frequency of some mERGs (Figures 5C–5F). To quantify the performance, we calculated the ratio of "positive" recording electrodes, where the peak power corresponding to each stimulation frequency exceeded the threshold (>4 SD), to "negative" recording electrodes, where the peak power corresponding to each stimulation frequency was below the threshold, in the grafted area of TP-*rd1* retinas and WT retinas (Figures 5G–5I).

Under the dark background condition, the ratio of positive to negative electrodes at a light intensity of 15.32 R*/rod/s was significantly lower than that in WT retinas at all frequencies, but approximately 50% of the electrodes followed the 5-Hz flicker stimulation (Figure 5G). When the light intensity was increased to 95.23 R*/rod/s, approximately 20% of the electrodes in the grafted area of TP-*rd1* retinas could follow the 15-Hz flicker stimulation, although the ratio of positive to negative electrodes in response to flicker stimulation at 15–25 Hz was significantly lower in TP-*rd1* retinas than that in WT retinas (Figure 5H).

Under the light-adapted condition (1,189.59 R*/rod/s background), the ratio of positive to negative electrodes at 15- to 25-Hz flicker stimulation of 43,944.81 R*/rod/s was significantly lower in TP-*rd1* retinas than that in WT retinas, but approximately 20% of the electrodes on the grafted area followed the 15-Hz flicker stimulation (Figure 5I). These results show that the TP-*rd1* retinas partially recovered temporal resolution to mesopic light stimuli under dark background conditions and photopic light stimuli under bright background conditions (Figures 5G–5I). Furthermore, similar to WT retinas, temporal resolution improved as the light intensity increased.

Next, we analyzed RGC firing responses to flicker stimuli in TP-*rd1* and WT retinas. Some RGCs in TP-*rd1* retinas responded to flicker stimuli (Figures 6A–6D). Autocorrelation calculated from the spike raster revealed periodic responses (Figures 6E–6H). The peak frequency of the power spectrum corresponded to the stimulus frequency in some RGCs (Figures 6I–6L). To quantify the performance, we calculated the ratio of "positive" RGCs, where the peak power corresponding to each stimulation frequency exceeded the threshold (>4 SD), to "negative" RGCs, where the peak power corresponding to each stimulation frequency was below the threshold, in ON, OFF, and ON-OFF RGCs of TP-*rd1* and WT retinas (Figures 6M–6O). For most stimulus conditions, the ratio of positive to negative RGCs was

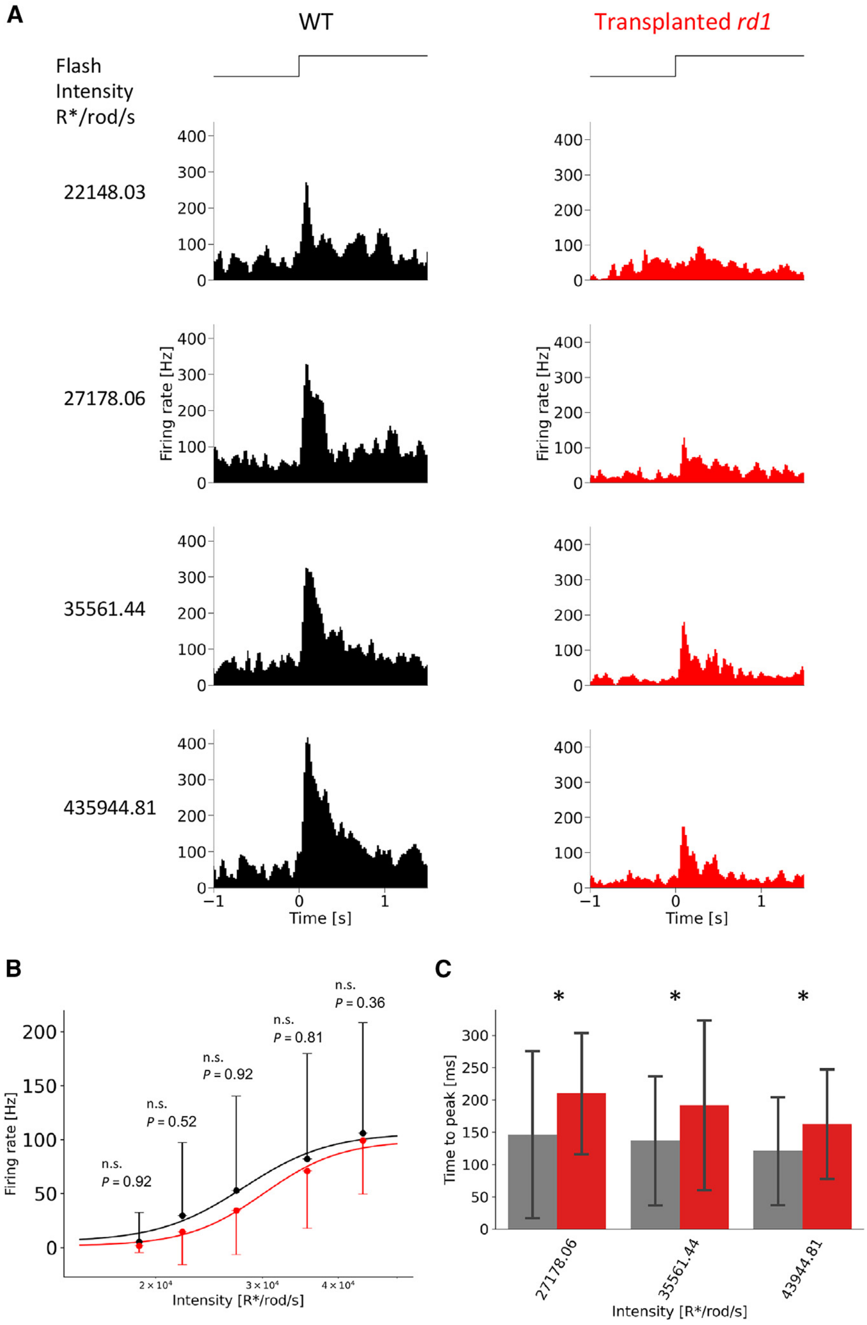

Figure 4. Adaptation to the photopic background light in the TP-*rd1* retina

(A) Representative light responses of RGCs in WT (black) and TP-*rd1* (red) retinas to flashes under the photopic background condition (15,441.33 R*/rod/s).

(B) Relationship between mean firing rate of ON peak responses and light intensity in WT (black, n = 68 cells from 3 retinas, 3 animals) and TP *rd1* (red, n = 16 cells from 3 retinas, 3 animals) retinas. Error bars denote standard deviation.

(C) Relationship between time-to-peak latency of ON responses and light intensity in WT (gray) and TP-*rd1* (red) retinas (27,178.06 R*/rod/s; p values = 0.015, WT: n = 38, TP-*rd1*: n = 8, 35,561.44 R*/rod/s; p = 0.044, WT: n = 51, Tp-*rd1*: n = 12, 43,944.81 R*/rod/s; p = 0.015, WT: n = 68, TP-*rd1*: n = 16, Mann-Whitney U test). Error bars denote standard deviation.

significantly lower in TP-*rd1* retinas than that in WT retinas (Figures 6M–6O), but approximately 5% of RGCs followed the 5-Hz flicker stimulation at a stimulus light intensity of 15.323 R*/rod/s and 43,944.81 R*/rod/s under the 1,189.59 R*/rod/s background light condition. Furthermore, approximately 35% of RGCs of TP-*rd1* retinas could follow the 5-Hz flicker stimulation at a light intensity of 95.23 R*/rod/s (Figure 6N). Therefore, the temporal frequency characteristics of the grafted area in the TP-*rd1* retina were partially recovered. Since RGCs are the final output neurons of the retina, it is highly likely that the transplantation of gRO sheets into the *rd1* retina may restore the ability to process and transmit high-frequency light changes to higher-order visual areas.

DISCUSSION

In gRO sheets with *Islet1* deletion, the number of ON BCs, including rod BCs, was significantly reduced, and the

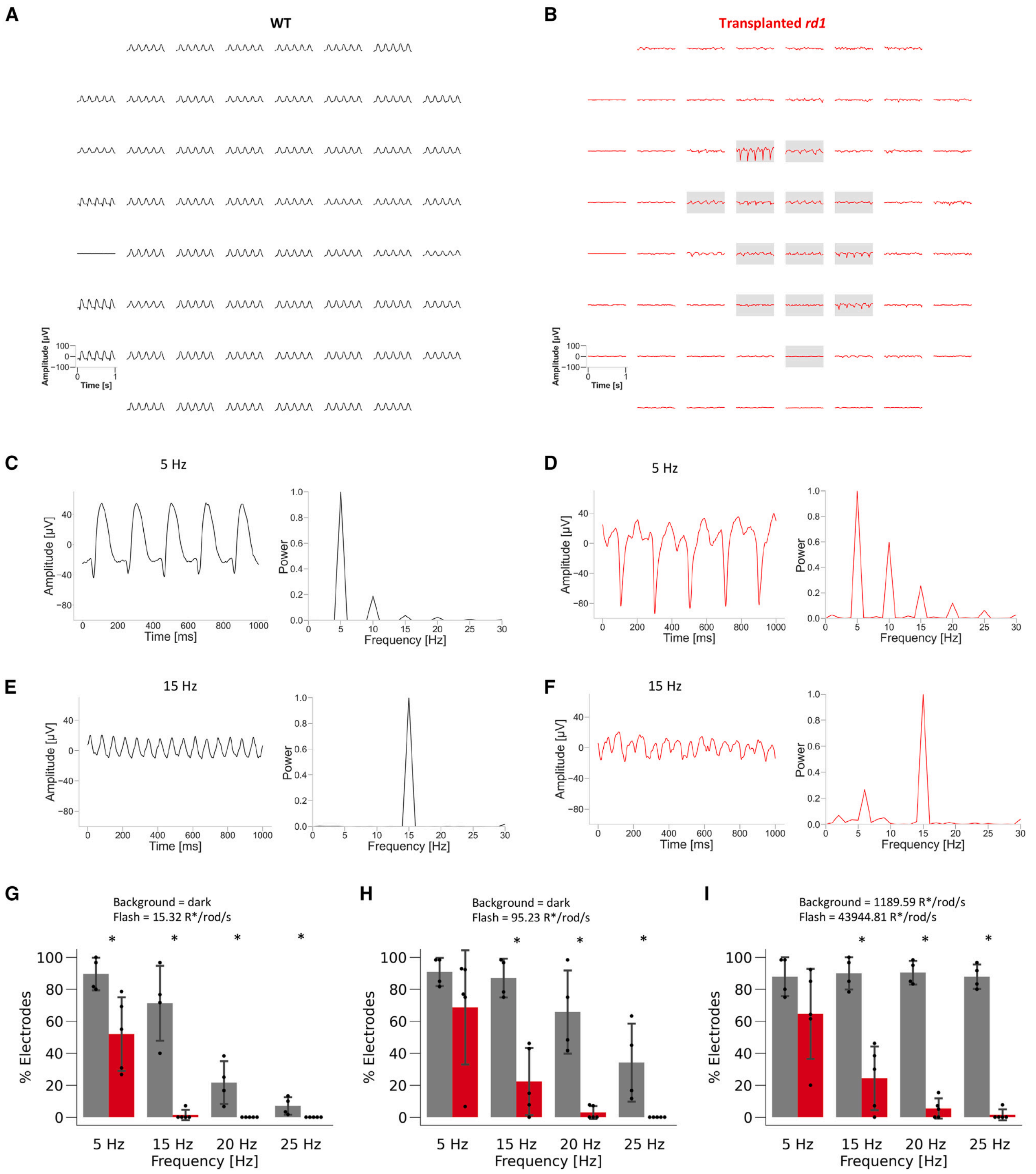

Figure 5. mERGs recorded from WT and TP-*rd1* retinas in response to flickering stimuli

(A and B) Representative mERGs during the 5-Hz flicker stimulation (95.23 R*/rod/s) recorded from WT (A) and TP-*rd1* (B) retinas by MEA. The waveform is low pass filtered at 100 Hz. Gray box indicates the electrodes under the grafted area (area 1).

(C and D) Representative responses (black squares shown in A) to the 5-Hz flicker stimulation (95.23 R*/rod/s) and their power spectrums obtained from WT (C) and TP-*rd1* (D) retinas.

(legend continued on next page)

transplantation of the gRO sheets into the *rd1* mouse retina improved synaptic connectivity between BCs in the host retina and photoreceptors in the grafted retina, resulting in an improvement in the signal-to-noise ratio and light responsiveness of RGCs (Matsuyama et al., 2021; Yamasaki et al., 2022). Based on the practicality and safety of using iPSC-derived RO sheets in patients in our previous clinical study (Hirami et al., 2023), our next step was to use these gRO sheets to maximize the potency of regenerative therapy. In this context, we characterized the properties of TP-*rd1* retinas, where *Islet1*$^{-/-}$ gRO sheets were transplanted into advanced end-stage retinal degeneration (*rd1*) mice aged 10–18 weeks. The most prominent feature in this study was that although the *rd1* retinas showed some remaining response to high-intensity light at 4–6 weeks of age, the older TP-*rd1* retinas newly acquired light responsiveness to scotopic-mesopic light stimulation (Figure 3). Furthermore, TP-*rd1* retinas adapted to photopic background light (Figure 4), although the time-to-peak latency of TP-*rd1* RGCs was significantly higher than that of WT RGCs, both in darkness and under bright background light. TP-*rd1* retinas could follow a flickering light stimulus, although the time resolution and time-to-peak latency were somewhat incomplete compared to those of WT retinas; for flicker stimulation at 15 Hz or higher under any of the background conditions, the number of positive electrodes and responding RGCs was significantly lower in TP-*rd1* retinas than in WT retinas (Figures 5 and 6). These results imply that the transplantation of gRO sheets has the potential to restore some functional properties by using the remaining network in severely degenerated retinas.

The mouse retina transmits ON and OFF information to the same extent (Goetz et al., 2022; Seifert et al., 2023). In this study, the PSTH of the spikes obtained from each electrode often exhibited both ON and OFF responses (Figures 2, S2, and S3). However, after performing spike sorting and confirming the presence of a refractory period (±1 ms) through autocorrelation analysis of the sorted spikes from each unit, we obtained reliable responses from a limited number of RGCs, the majority of which were of the ON types with less ON-OFF types in the TP-*rd1* retinas, resulting in a decrease of overall OFF signal transmission (Figures 2, S2, and S3). Although we cannot exclude the possibility that OFF-type responses were either missed during spike sorting or classified as “low signal” in the current analysis, the ratio of OFF-type to ON-type RGCs in the transplanted retinas was consistently low in our previous study (Matsuyama et al., 2021). This suggests that ON and OFF information may be processed differently in degenerating and transplanted retinas. In retinas with degenerated photoreceptors, the photoreceptors and BCs form new synaptic connections and alter their receptor expression patterns (D’Orazi et al., 2014). In *rd10* mouse retinas, the OFF pathway was less excitable due to an increase in presynaptic inhibition (Carleton and Oesch, 2024). Another possibility is that synaptic remodeling may be biased toward the ON pathway in TP-*rd1* retinas. We have confirmed the synaptic connectivity between the rod photoreceptors in the grafted gRO sheets and BCs in the degenerated host retina using reporter mice (*L7*-GFP) and synapse reporter grafts (*Nrl*-CtBP2:tdTomato), with additional immunostaining of postsynaptic markers (Figure 1). The identification of synapses between photoreceptors in the grafted gRO sheets and OFF BCs in the host *rd1* retina may help to understand the possible bias in ON and OFF pathway reconstruction during regenerative therapy.

The retina is a sophisticated system that senses changes in light intensity over a wide range of background light intensities. As the stimulus intensity increased in the scotopic-mesopic range, the dark-adapted TP-*rd1* retinas showed an increase in mean response intensity and a decrease in time-to-peak latency similar to WT retinas (Figure 3). Furthermore, RGCs of TP-*rd1* retinas also adapted to photopic background light and responded to incremental light stimuli (Figure 4). These results suggest that signals from photoreceptors in the gRO sheets coordinate with the host retinal network to function properly under dark- and light-adapted conditions. However, the time-to-peak latency of RGC responses in TP-*rd1* retinas was significantly delayed compared to that in WT retinas. These characteristics are similar to those of human iPS-derived cones *in vitro* (Saha et al., 2022). As photoreceptor ribbon synapses have a complex structure, it seems likely that the transmission efficiency from photoreceptors in the grafted retina to BCs in the host retina may vary depending on the status of each *de novo* synapse. Recently, we have reported that host-graft synapses identified by *L7*-GFP and *Nrl*-CtBP2:tdTomato may display invagination of horizontal

(E and F) Representative responses (black squares shown in A) to the 15-Hz flicker stimulation (43,944.81 R*/rod/s) under the 1,189.59 R*/rod/s background light and their power spectrums obtained from WT (E) and TP-*rd1* (F) retinas.
(G–I) Percentage of electrodes that showed mERGs following flickering stimulation in WT (black, 4 retinas, 4 animals) and TP-*rd1* (red, 5 retinas, 5 animals) retinas. In (G), the stimulus condition was 15.32 R*/rod/s under the dark background (5 Hz; $p = 0.016$, 15 Hz; $p = 0.015$, 20 Hz, $p = 0.011$, 25 Hz; $p = 0.011$, Mann-Whitney U test). In (H), the stimulus condition was 95.23 R*/rod/s under the dark background (5 Hz; $p = 0.18$, 15 Hz; $p = 0.016$, 20 Hz; $p = 0.018$, 25 Hz; $p = 0.011$, Mann-Whitney U test). In (I), the stimulus condition was 43,944.81 R*/rod/s under the 1,189.59 R*/rod/s background. (5 Hz; $p = 0.18$, 15 Hz; $p = 0.016$, 20 Hz; $p = 0.019$, 25 Hz; $p = 0.015$, Mann-Whitney U test). Error bars denote standard deviation.

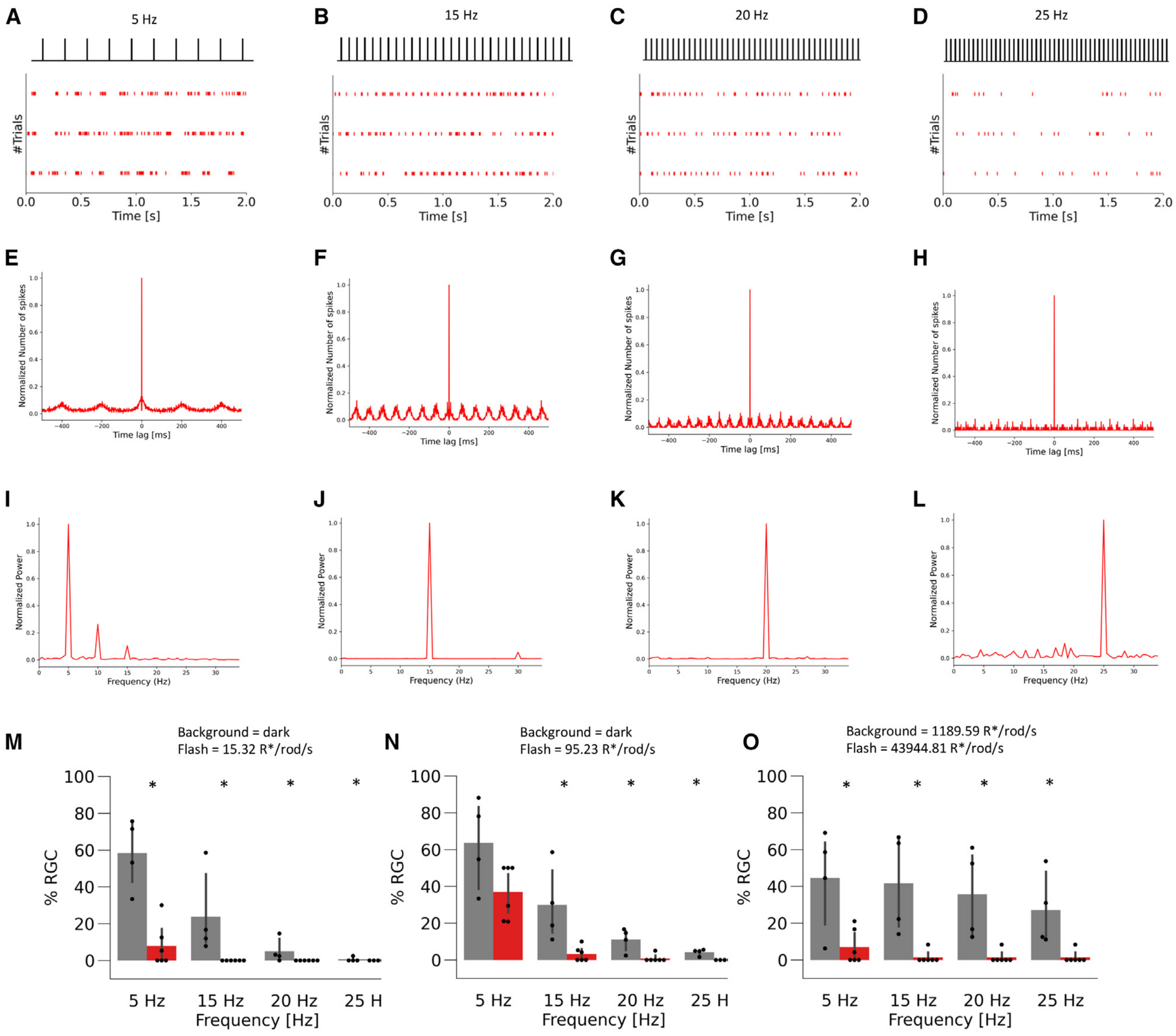

Figure 6. RGC firing responses to flickering stimulation in the TP-*rd1* retina

(A–D) Representative spike raster of an RGC in the TP-*rd1* retina in response to 5–25 Hz flickering stimulation (43944.81 R*/rod/s) under the 1,189.59 R*/rod/s background light.

(E–H) The auto-correlograms calculated from the responses shown in (A)–(D).

(I–L) The power spectrums calculated from the auto-correlograms shown in (E)–(H).

(M–O) Ratio of positive to negative RGCs for each flickering stimulation in WT (black, 165 RGCs, 4 retinas, 4 animals) and TP-*rd1* (red, 98 RGCs, 5 retinas, 5 animals) retinas. In (M), the stimulus condition was 15.32 R*/rod/s under the dark background (5 Hz; $p = 0.013$, 15 Hz; $p = 0.0057$, 20 Hz; $p = 0.026$, 25 Hz; $p = 0.31$ Mann-Whitney U test). In (N), the stimulus condition was 95.23 R*/rod/s under the dark background (5 Hz; $p = 0.067$, 15 Hz; $p = 0.013$, 20 Hz; $p = 0.017$, 25 Hz; $p = 0.0057$, Mann-Whitney U test). In (O), the stimulus condition was 43,944.81 R*/rod/s under the 1,189.59 R*/rod/s background light (5 Hz; $p = 0.040$, 15 Hz; $p = 0.0089$, 20 Hz; $p = 0.0089$, 25 Hz; $p = 0.0089$, Mann-Whitney U test). Error bars denote standard deviation.

cell processes and host BC dendrites toward the grafted photoreceptor ribbons with a "fluffy density" that indicates the presence of a complex of synaptic molecules (Akiba et al., 2024). Although we did not confirm the microstructure of host-graft synapses with gROs in this study, the presence of mGluR6 at the synaptic site may suggest the presence of a similar synaptic structure, which we plan to investigate in detail in future studies. Additionally, light adaptation does not occur normally in horizontal cell (HC)-deleted mouse retinas (Chaya et al., 2017). OFF

αRGCs of mouse retinas with HC-specific deletion of GluA2/4 are defective in tuning to spatiotemporal frequency and contrast (Ströh et al., 2018). HCs are present in both *rd1* mouse retina and gRO grafts (Matsuyama et al., 2021; Yamasaki et al., 2022), but it is not yet clear how HCs are involved in the process of synapse formation between photoreceptors in the grafted retina and BCs in the host retina. Another unique functional property of the retina is temporal resolution, which changes depending on the light intensity (Pasquale et al., 2020). TP-*rd1* retinas also adapted to background light and showed temporal resolution to flickering stimuli, similar to WT retinas. However, the number of RGCs that followed high-frequency stimuli was significantly smaller in TP-*rd1* retinas than in WT retinas, indicating that rod reconstruction might be dominant. Interestingly, the shape of the mERG waveform in TP-*rd1* retinas was different from that in WT retinas and varied from one electrode to another (Figure 5), reflecting the variable status of the gRO sheets at each electrode. ERG is a mass-field potential change that originates from extracellular currents generated by each retinal component in response to light stimulation (Perlman, 2015). Since photoreceptors in RO grafts often form a rosette-like morphology, the dark current along the photoreceptor outer segments may flow in variable directions, resulting in an a-wave with a smaller amplitude than that through normally aligned photoreceptors. The inefficient recycling of visual pigments can also affect the temporal resolution of TP-*rd1* retinas because photoreceptors in a rosette may be easily saturated due to poor interaction with the retinal pigment epithelium.

RGC responses were occasionally detected outside of the grafted areas (Figure 2). In our previous report, we observed that *Islet1*$^{-/-}$ gRO signals were more far-reaching than non-gRO signals (Matsuyama et al., 2021). Additionally, we recently observed that the RGC responses were highly correlated with the number of host-graft synapses located within a 100 ± 25 μm radius from the electrodes (Akiba et al., 2024). A subtype of RGCs that responds to light even with spot stimuli of approximately 500 μm or larger (Wienbar and Schwartz, 2018) has been reported. Therefore, some RGCs outside the grafted area may receive signals from the grafted photoreceptor cells, in addition to a possibility that they may receive some signals from a few remaining photoreceptors.

Material transfer from the photoreceptors in the graft to the remaining host photoreceptors is a matter of debate in these studies (Pearson et al., 2016; Santos-Ferrieira et al., 2016; Singh et al., 2016; Decembrini et al., 2017; Ortin-martinez et al., 2017). However, as material transfer has been shown to occur primarily between photoreceptors, this seems unlikely because we used late-stage *rd1* mice with photoreceptor degeneration. Evidently, transplantation of *Islet1*$^{-/-}$ gRO sheets into advanced *rd1* mice substantially recovered, or even newly acquired, light-evoked responses that were absent in the much younger *rd1* mice. This also implies that improved retinal sensitivity can be a useful parameter for substantial functional integration of *Islet1*$^{-/-}$ gRO sheets in future clinical evaluation. In conclusion, our results suggest that *Islet1*$^{-/-}$ gRO sheets are promising candidates for use in regenerative cell therapy for human advanced retinal degeneration.

Limitations

In this study, we applied a strict spike-sorting protocol, which resulted in a limited number of RGCs for the analysis. However, we obtained a substantial number of RGCs to compare their characteristics among the WT, TP-*rd1*, and *rd1* retinas. Electrophysiological analyses revealed that some retinal functions were restored in the transplanted retinas. However, we did not conduct behavioral tests using different types of visual stimuli; therefore, whether the consequences of these electrophysiological results are related to subjective vision remains unclear. Additionally, the current analysis did not include parameters related to spatial resolution, such as receptive fields, which can be estimated using pattern stimulation devices to conduct random checkerboard or grating stimulations. Furthermore, a detailed investigation of the histological parameters that may correlate with electrophysiological outcomes may also lead to new challenges and potential improvements in regenerative cell therapy for human patients.

EXPERIMENTAL PROCEDURES

Animals

All experimental protocols were approved by the animal care committee of the RIKEN Center for Biosystems Dynamics Research and were conducted in accordance with local guidelines and the ARVO statement on the use of animals in ophthalmic and vision research.

The progressive retinal degeneration mouse line C57BL/6J-Pde6b^{rd1-2J}/J (JAX stock #004766, referred to as *rd1*) was crossed with B6; FVB-Tg(Pcp2-EGFP)2Yuza/J (JAX stock #004690) (Tomomura et al., 2001) to produce *rd1*;*L7*-GFP mice expressing EGFP in rod BCs. Mouse gRO sheets were transplanted subretinally into 10- to 31-week-old *rd1*;*L7*-GFP mice (n = 7). Six TP-*rd1* mice were used in this experiment (Table S1). Additionally, C57BL/6J mice (referred to as WT, n = 6) and 4- to 6-week *rd1* (n = 6) were used in the experiment.

Mouse ES cell line and retinal organoid differentiation

The mouse ES cell line ROSA26$^{+/Nrl-\text{CtBP2:tdTomato}}$ is a synaptic ribbon reporter line that expresses the CtBP2:tdTomato fusion protein under the control of the *Nrl* promoter (Mandai et al., 2017). We prepared retinal sheets for transplantation from the *Islet1*$^{-/-}$

ROSA26$^{+/Nrl\text{-CtBP2:tdTomato}}$ mouse ES line (Matsuyama et al., 2021). Differentiation protocol and transplantation procedure is described in supplemental procedures.

Electrophysiology: MEA recordings

Host retinas with the engrafted gRO sheets were isolated for MEA recordings ≥6 weeks after transplantation. The following procedure was performed under a dim LED light with a peak wavelength of 690 nm. After a day of dark adaptation, the mice were anesthetized with isoflurane (VIATRIS) and sacrificed by cervical dislocation. After enucleation, the cornea and vitreous were removed, and only the retina was isolated. The transplant was determined visually. The retina was trimmed around the engrafted area to an appropriate size and placed, RGC side down, onto the MEA electrodes (Multi Channel Systems, 60pMEA200/30iR-Ti: 60 electrodes, electrode size 30 × 30 μm, inter-electrode distance 200 μm). The flat-mounted retina was fixed on the electrodes by suction using a vacuum pump, and the retina was constantly perfused with warmed (34°C), carbonated (95% O_2 and 5% CO_2) Ames' medium (Sigma, A1420) at 3–3.5 mL/min. Recordings were started 40 min after perfusion initiation. Extracellular voltage signals were amplified and digitized at 20 kHz using an MEA amplifier (Multi Channel Systems, USB-ME64-System).

Immunohistochemistry

Details of immunocytochemistry and antibodies used are described in the supplemental procedures.

Light simulation for MEA recordings

The light stimulus was generated using an LED light with a single peak at 505 nm (Thorlabs, SOLIS-505C), and the entire retina was illuminated through an objective lens. The light intensity was adjusted using an LED modulator (Thorlabs, DC2200) controlled by a function generator (NF Corporation, WF1973). Details of light simulation for MEA recordings are described in the supplemental procedures.

Data analysis

Details of spike sorting are described in the supplemental procedures. We divided the MEA area into four groups (areas 1–4) according to the distance from the grafted area, which was identified by the presence of CtBP2:tdTomato fluorescence expressed at the photoreceptor terminals of the graft. Area 1 corresponded to the graft and areas 2–4 referred to areas that gradually expanded away from the graft. To label the regions within the graft and its surrounding area based on their proximity, we applied binary dilation at magnifications of 1×, 5×, 10×, and 15×. This approach allowed us to systematically dilate the labeled regions according to their shapes with different grafts, facilitating spatial analysis of the tissue. PSTHs were calculated with a 20-ms bin width and passed through a binomial filter ($n = 4$) for smoothing. Based on the shape of the response to a 2-s flash (95.23 R*/rod/s), each RGC was classified as ON, OFF, ON-OFF, low signal, or not classified. The mean firing rate was calculated from spontaneous activity over 10 s in the dark. The threshold was calculated as the mean firing rate +4 SD. The response peak was corrected by subtracting the mean firing rate in the dark. When the response peak (>threshold) was detected within 500 ms after light onset, the RGC was classified as ON. When the response peak (>threshold) was detected 50–300 ms after light offset, it was classified as OFF. When response peaks appeared at both light onset and offset, it was classified as ON-OFF. When the peak firing rates (>threshold) were <10 Hz, it was classified as low signal. RGCs that were not identified as any type were not classified. In Figure 3, the analyzed data include both ON and ON-OFF types. In Figure 3C, the subthreshold response was 0 Hz.

The intensity response curve was fitted using the following equation (Westö et al., 2022):

$$Fit = Rmin + (Rmax - Rmin)\frac{I^n}{I^n + I_{50}{}^n}$$

Rmin and *Rmax* are the smallest and largest peak firing rates, respectively, among all the stimulus intensities. I_{50} is the intensity at the half-maximum response. I_{50} and n values were determined by minimizing the squared error between the measured data and predicted values of the function.

To process the flickering ERGs, the raw waveform was passed through a 100-Hz low-pass filter, and three waveforms under the same stimulus condition were averaged. To evaluate the flicker frequency, the power spectrum was calculated from the waveform (scipy.signal.periodgram, scipy v.1.9.1). The mean power from 0 to 30 Hz was calculated from the 1-s average waveform in the dark. Each electrode was considered as "positive" or "negative" when its power at each stimulation frequency was above or below the mean +4 SD. To quantify the performance, we calculated the ratio of "positive" to "negative" electrodes.

To analyze RGC responses to flicker stimulation, autocorrelation was calculated from the raster. The power spectrum was then calculated from the autocorrelation (scipy.signal.periodgram, scipy v.1.9.1). The average power from 0 to 30 Hz was calculated from the average waveform in response to each stimulation. An RGC was considered "positive" or "negative" when its power at each stimulation frequency was above or below the mean +4 SD. To quantify the performance, we calculated the ratio of "positive" to "negative" RGCs.

Statistical analysis

In Figures 3D, 4B, 4C, 5G, 5H, 5I, 6M, 6N, and 6O, the Mann-Whitney U test was used. In Figure 3E, the Kolmogorov-Smirnov test was used. Error bars denote standard deviation. The following asterisks in the figures indicate p values: * ≤ 0.05, **≤ 0.01, ***≤ 0.001.

RESOURCE AVAILABILITY

Lead contact

Further information and requests for resources and reagents should be directed to the corresponding author, Michiko Mandai (e_lab.mandai@kcho.jp).

Materials availability

This study did not generate new unique reagents.

Data and code availability

The datasets supporting the current study have not been deposited in a public repository because of the large data size but are

available from the corresponding author upon request. Any additional information required to reanalyze the data reported in this paper is available from the corresponding author upon reasonable request.

ACKNOWLEDGMENTS

We thank Junki Sho, Chihiro Hayakawa, Hironobu Syuto, and Toshika Senba for support with animal experiments. This study was supported by JSPS KAKENHI 22K09826. We thank Dr. Jeannie Chen (University of Southern California) for providing mGluR6 antibody.

AUTHOR CONTRIBUTIONS

Conceptualization, M.W., M. Tachibana, and M.M.; data curation, M.W.; formal analysis, M.W.; funding acquisition, M.M.; investigation, M.W. and T.Y.; methodology, M.W., M. Tachibana., and M.M.; project administration, M.M.; resources, M.M.; supervision, C.K., M. Tachibana, M. Takahashi, and M.M.; visualization, M.W.; writing – original draft, M.W., M. Tachibana, and M.M.

DECLARATION OF INTERESTS

M. Takahashi is a founder of Vision Care, Inc. M.W. is an employee of VCCT, Inc. T.Y. is an employee of Vision Care, Inc. M.M. and M. Takahashi are co-inventors on patent application regarding the genetically modified retinal organoids.

SUPPLEMENTAL INFORMATION

Supplemental information can be found online at https://doi.org/10.1016/j.stemcr.2024.102393.

Received: July 17, 2024
Revised: December 18, 2024
Accepted: December 18, 2024
Published: January 16, 2025

REFERENCES

Akiba, R., Tu, H.Y., Hashiguchi, T., Takahashi, Y., Toyooka, K., Tsukamoto, Y., Baba, T., Takahashi, M., and Mandai, M. (2024). Host graft synapses form functional microstructures and shape the host light responses after stem cell derived retinal sheet transplantation. Invest. Ophthalmol. Vis. Sci. *65*, 8. https://doi.org/10.1167/iovs.65.12.8.

Ayton, L.N., Barnes, N., Dagnelie, G., Fujikado, T., Goetz, G., Hornig, R., Jones, B.W., Muqit, M.M.K., Rathbun, D.L., Stingl, K., et al. (2020). An update on retinal prostheses. Clin. Neurophysiol. *131*, 1383–1398. https://doi.org/10.1016/j.clinph.2019.11.029.

Bi, A., Cui, J., Ma, Y.P., Olshevskaya, E., Pu, M., Dizhoor, A.M., and Pan, Z.H. (2006). Ectopic expression of a microbial-type rhodopsin restores visual responses in mice with photoreceptor degeneration. Neuron *50*, 23–33. https://doi.org/10.1016/j.neuron.2006.02.026.

Biswas, S., Haselier, C., Mataruga, A., Thumann, G., Walter, P., and Müller, F. (2014). Pharmacological analysis of intrinsic neuronal oscillations in rd10 retina. PLoS One *9*, e99075. https://doi.org/10.1371/journal.pone.0099075.

Botto, C., Rucli, M., Tekinsoy, M.D., Pulman, J., Sahel, J.A., and Dalkara, D. (2022). Early and late stage gene therapy interventions for inherited retinal degenerations. Prog. Retin. Eye Res. *86*, 100975. https://doi.org/10.1016/j.preteyeres.2021.100975.

Carleton, M., and Oesch, N.W. (2024). Asymmetric activation of ON and OFF pathways in the degenerated retina. eNeuro *11*. ENEURO.0110-24.2024. https://doi.org/10.1523/ENEURO.0110-24.2024.

Chaya, T., Matsumoto, A., Sugita, Y., Watanabe, S., Kuwahara, R., Tachibana, M., and Furukawa, T. (2017). Versatile functional roles of horizontal cells in the retinal circuit. Sci. Rep. *7*, 5540. https://doi.org/10.1038/s41598-017-05543-2.

D'Orazi, F.D., Suzuki, S.C., and Wong, R.O. (2014). Neuronal remodeling in retinal circuit assembly, disassembly, and reassembly. Trends Neurosci. *37*, 594–603. https://doi.org/10.1016/j.tins.2014.07.009.

Decembrini, S., Martin, C., Sennlaub, F., Chemtob, S., Biel, M., Samardzija, M., Moulin, A., Behar-Cohen, F., and Arsenijevic, Y. (2017). Cone genesis tracing by the Chrnb4-EGFP mouse line: evidences of cellular material fusion after cone precursor transplantation. Mol. Ther. *25*, 634–653. https://doi.org/10.1016/j.ymthe.2016.12.015.

Fujii, M., Sunagawa, G.A., Kondo, M., Takahashi, M., and Mandai, M. (2016). Evaluation of micro electroretinograms recorded with multiple electrode array to assess focal retinal function. Sci. Rep. *6*, 30719. https://doi.org/10.1038/srep30719.

Goetz, J., Jessen, Z.F., Jacobi, A., Mani, A., Cooler, S., Greer, D., Kadri, S., Segal, J., Shekhar, K., Sanes, J.R., and Schwartz, G.W. (2022). Unified classification of mouse retinal ganglion cells using function, morphology, and gene expression. Cell Rep. *40*, 111040. https://doi.org/10.1016/j.celrep.2022.111040.

Hirami, Y., Mandai, M., Sugita, S., Maeda, A., Maeda, T., Yamamoto, M., Uyama, H., Yokota, S., Fujihara, M., Igeta, M., et al. (2023). Safety and stable survival of stem-cell-derived retinal organoid for 2 years in patients with retinitis pigmentosa. Cell Stem Cell *30*, 1585–1596.e6. https://doi.org/10.1016/j.stem.2023.11.004.

Jones, B.W., Pfeiffer, R.L., Ferrell, W.D., Watt, C.B., Marmor, M., and Marc, R.E. (2016). Retinal remodeling in human retinitis pigmentosa. Exp. Eye Res. *150*, 149–165. https://doi.org/10.1016/j.exer.2016.03.018.

Kalloniatis, M., Nivison-Smith, L., Chua, J., Acosta, M.L., and Fletcher, E.L. (2016). Using the rd1 mouse to understand functional and anatomical retinal remodeling and treatment implications in retinitis pigmentosa: A review. Exp. Eye Res. *150*, 106–121. https://doi.org/10.1016/j.exer.2015.10.019.

Lagali, P.S., Balya, D., Awatramani, G.B., Münch, T.A., Kim, D.S., Busskamp, V., Cepko, C.L., and Roska, B. (2008). Light-activated channels targeted to ON bipolar cells restore visual function in retinal degeneration. Nat. Neurosci. *11*, 667–675. https://doi.org/10.1038/nn.2117.

Mandai, M. (2023). Pluripotent stem cell-derived retinal organoid/cells for retinal regeneration therapies: a review. Regen. Ther. *22*, 59–67. https://doi.org/10.1016/j.reth.2022.12.005.

Mandai, M., Fujii, M., Hashiguchi, T., Sunagawa, G.A., Ito, S.I., Sun, J., Kaneko, J., Sho, J., Yamada, C., and Takahashi, M. (2017). iPSC-derived retina transplants improve vision in rd1 end-stage retinal degeneration mice. Stem Cell Rep. *8*, 69–83. https://doi.org/10.1016/j.stemcr.2016.12.008.

Matsuyama, T., Tu, H.-Y., Sun, J., Hashiguchi, T., Akiba, R., Sho, J., Fujii, M., Onishi, A., Takahashi, M., and Mandai, M. (2021). Genetically engineered stem cell derived retinal grafts for improved retinal reconstruction after transplantation. iScience *24*, 102866. https://doi.org/10.1016/j.isci.2021.102866.

Ortin-Martinez, A., Tsai, E.L.S., Nickerson, P.E., Bergeret, M., Lu, Y., Smiley, S., Comanita, L., and Wallace, V.A. (2017). A reinterpretation of cell transplantation: GFP transfer from donor to host photoreceptors. Stem Cell. *35*, 932–939. https://doi.org/10.1002/stem.2552.

Pasquale, R., Umino, Y., and Solessio, E. (2020). Rod photoreceptors signal fast changes in daylight levels using a Cx36-independent retinal pathway in mouse. J. Neurosci. *40*, 796–810. https://doi.org/10.1523/JNEUROSCI.0455-19.2019.

Pearson, R.A., Gonzalez-Cordero, A., West, E.L., Ribeiro, J.R., Aghaizu, N., Goh, D., Sampson, R.D., Georgiadis, A., Waldron, P.V., Duran, Y., et al. (2016). Donor and host photoreceptors engage in material transfer following transplantation of post-mitotic photoreceptor precursors. Nat. Commun. *7*, 13029. https://doi.org/10.1038/ncomms13029.

Perlman, I. (2015). The electroretinogram: ERG by IDO Perlman. Webvision: The Organization of the Retina and Visual System. https://webvision.med.utah.edu/book/electrophysiology/the-electroretinogram-erg/

Saha, A., Capowski, E., Fernandez Zepeda, M.A., Nelson, E.C., Gamm, D.M., and Sinha, R. (2022). Cone photoreceptors in human stem cell-derived retinal organoids demonstrate intrinsic light responses that mimic those of primate fovea. Cell Stem Cell *29*, 460–471.e3. https://doi.org/10.1016/j.stem.2022.01.002.

Sahel, J.A., Boulanger-Scemama, E., Pagot, C., Arleo, A., Galluppi, F., Martel, J.N., Esposti, S.D., Delaux, A., de Saint Aubert, J.-B., de Montleau, C., et al. (2021). Partial recovery of visual function in a blind patient after optogenetic therapy. Nat. Med. *27*, 1223–1229. https://doi.org/10.1038/s41591-021-01351-4.

Sancho-Pelluz, J., Arango-Gonzalez, B., Kustermann, S., Romero, F.J., Van Veen, T., Zrenner, E., Ekström, P., and Paquet-Durand, F. (2008). Photoreceptor cell death mechanisms in inherited retinal degeneration. Mol. Neurobiol. *38*, 253–269. https://doi.org/10.1007/s12035-008-8045-9.

Santos-Ferreira, T., Llonch, S., Borsch, O., Postel, K., Haas, J., and Ader, M. (2016). Retinal transplantation of photoreceptors results in donor–host cytoplasmic exchange. Nat. Commun. *7*, 13028. https://doi.org/10.1038/ncomms13028.

Seifert, M., Roberts, P.A., Kafetzis, G., Osorio, D., and Baden, T. (2023). Birds multiplex spectral and temporal visual information via retinal On-and Off-channels. Nat. Commun. *14*, 5308. https://doi.org/10.1038/s41467-023-41032-z.

Singh, M.S., Balmer, J., Barnard, A.R., Aslam, S.A., Moralli, D., Green, C.M., Barnea-Cramer, A., Duncan, I., and MacLaren, R.E. (2016). Transplanted photoreceptor precursors transfer proteins to host photoreceptors by a mechanism of cytoplasmic fusion. Nat. Commun. *7*, 13537. https://doi.org/10.1038/ncomms13537.

Ströh, S., Puller, C., Swirski, S., Hölzel, M.-B., van der Linde, L.I.S., Segelken, J., Schultz, K., Block, C., Monyer, H., Willecke, K., et al. (2018). Eliminating glutamatergic input onto horizontal cells changes the dynamic range and receptive field organization of mouse retinal ganglion cells. J. Neurosci. *38*, 2015–2028. https://doi.org/10.1523/JNEUROSCI.0141-17.2018.

Tomomura, M., Rice, D.S., Morgan, J.I., and Yuzaki, M. (2001). Purification of Purkinje cells by fluorescence-activated cell sorting from transgenic mice that express green fluorescent protein. Eur. J. Neurosci. *14*, 57–63. https://doi.org/10.1046/j.0953-816x.2001.01624.x.

Trenholm, S., and Awatramani, G.B. (2015). Origins of spontaneous activity in the degenerating retina. Front. Cell. Neurosci. *9*, 277. https://doi.org/10.3389/fncel.2015.00277.

Tu, H.Y., Chen, Y.J., McQuiston, A.R., Chiao, C.C., and Chen, C.K. (2015). A novel retinal oscillation mechanism in an autosomal dominant photoreceptor degeneration mouse model. Front. Cell. Neurosci. *9*, 513. https://doi.org/10.3389/fncel.2015.00513.

Watari, K., Yamasaki, S., Tu, H.Y., Shikamura, M., Kamei, T., Adachi, H., Tochitani, T., Kita, Y., Nakamura, A., Ueyama, K., et al. (2023). Self-organization, quality control, and preclinical studies of human iPSC-derived retinal sheets for tissue-transplantation therapy. Commun. Biol. *6*, 164. https://doi.org/10.1038/s42003-023-04543-5.

Westö, J., Martyniuk, N., Koskela, S., Turunen, T., Pentikäinen, S., and Ala-Laurila, P. (2022). Retinal OFF ganglion cells allow detection of quantal shadows at starlight. Curr. Biol. *32*, 2848–2857.e6. https://doi.org/10.1016/j.cub.2022.04.092.

Wienbar, S., and Schwartz, G.W. (2018). The dynamic receptive fields of retinal ganglion cells. Prog. Retin. Eye Res. *67*, 102–117. https://doi.org/10.1016/j.preteyeres.2018.06.003.

Yamasaki, S., Tu, H.-Y., Matsuyama, T., Horiuchi, M., Hashiguchi, T., Sho, J., Kuwahara, A., Kishino, A., Kimura, T., Takahashi, M., and Mandai, M. (2022). A Genetic modification that reduces ON-bipolar cells in hESC-derived retinas enhances functional integration after transplantation. iScience *25*, 103657. https://doi.org/10.1016/j.isci.2021.102866103657.

Stem Cell Reports, Volume *20*

Supplemental Information

Transplantation of genome-edited retinal organoids restores some fundamental physiological functions coordinated with severely degenerated host retinas

Mikiya Watanabe, Takayuki Yamada, Chieko Koike, Masayo Takahashi, Masao Tachibana, and Michiko Mandai

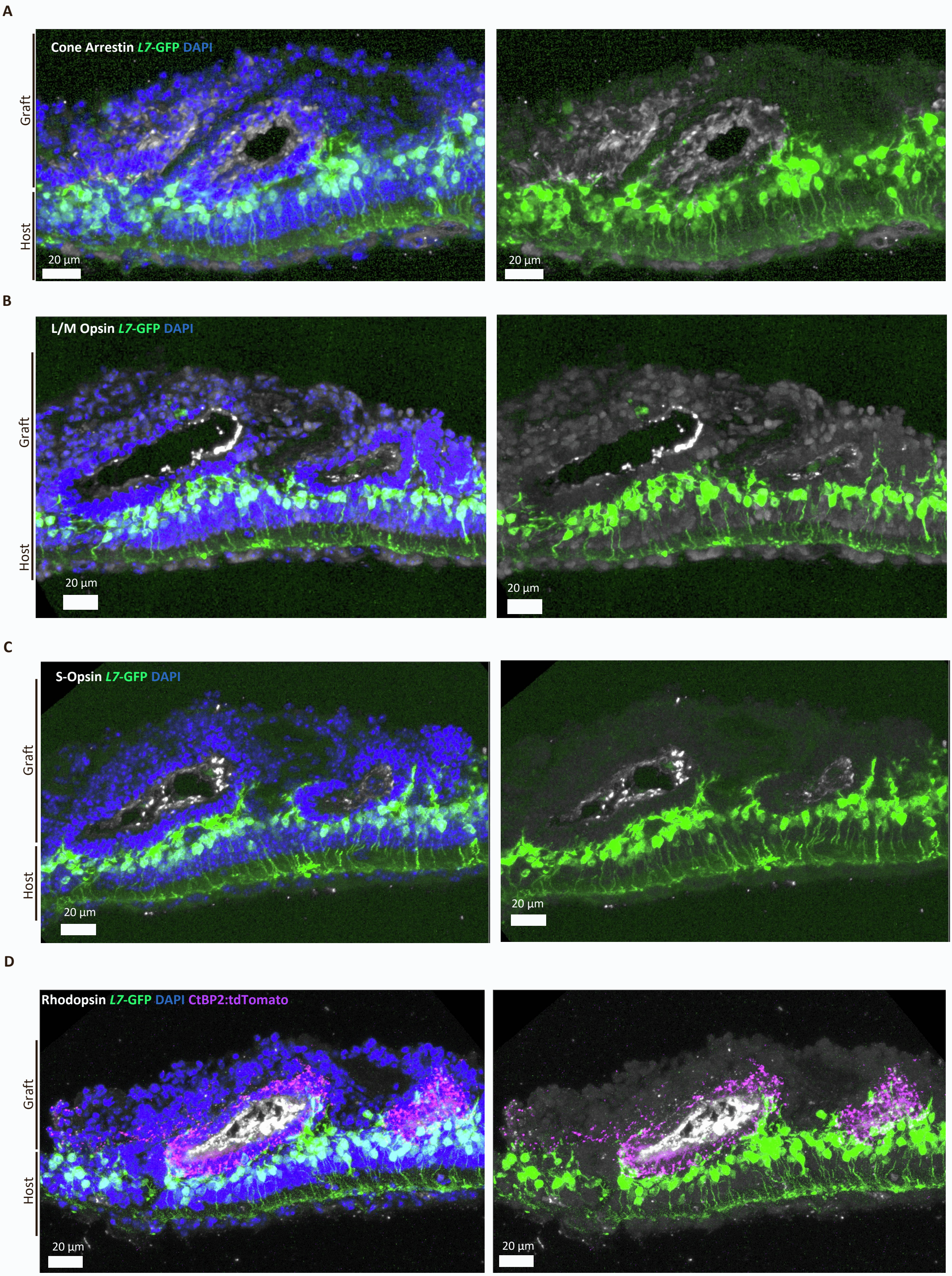

Figure S1. Immunostaining of TP-*rd1* retinal sections

(A-D) Immunostaining for TP-*rd1* retina with cone arrestin, L/M opsin, S-opsin and rhodopsin.

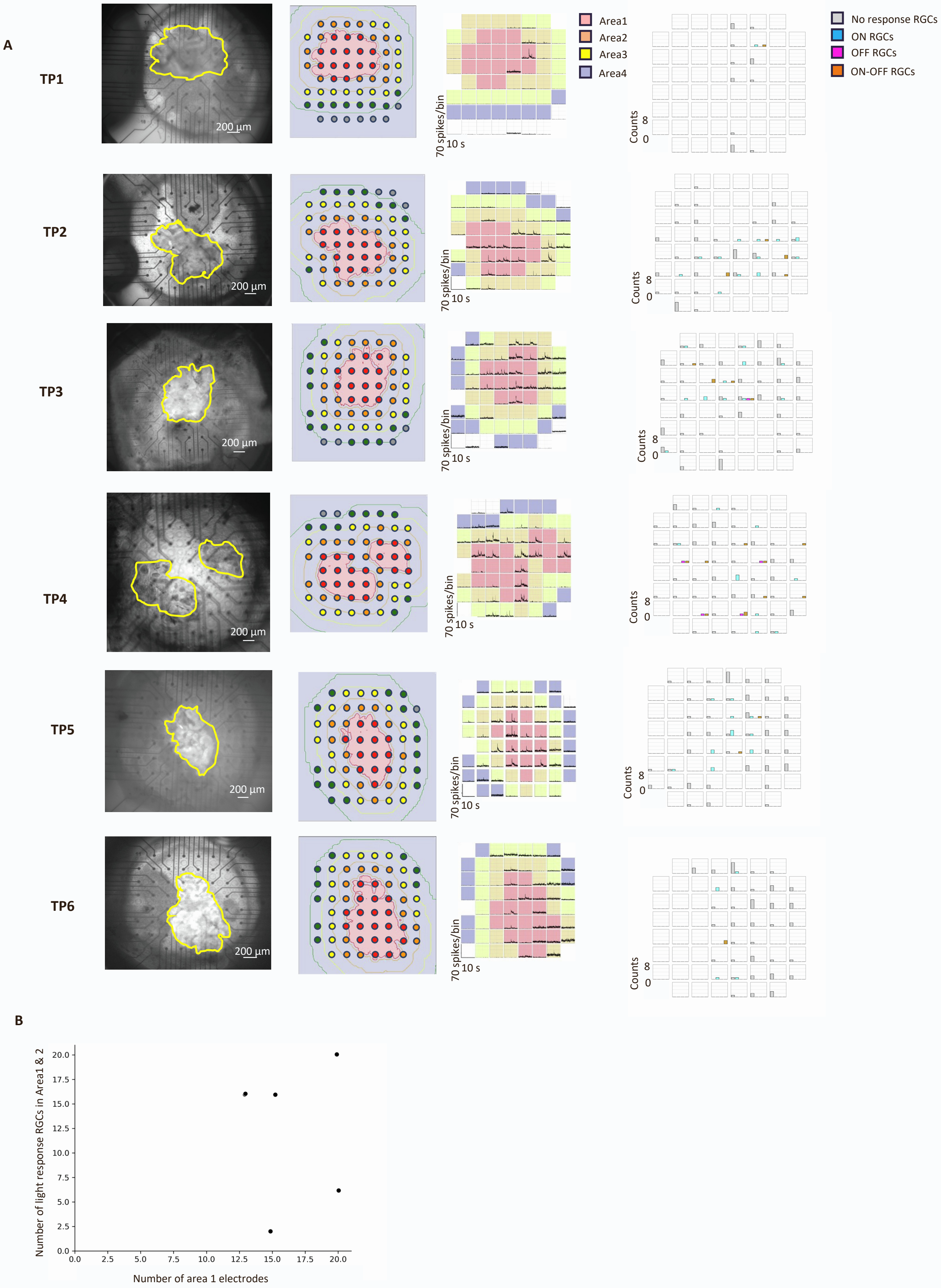

Figure S2. Summary of each TP-*rd1* retina recorded by MEA

(A)Photographs of the retina with CtBP2:tdTomato fluorescence (except for TP4 with Infra-Red image) during MEA recordings for each TP-*rd1*, along with mapping results and the time histogram of spikes

(B) Relationship between the number of light responses RGCs in area 1-2 and the number of electrodes in area 1.

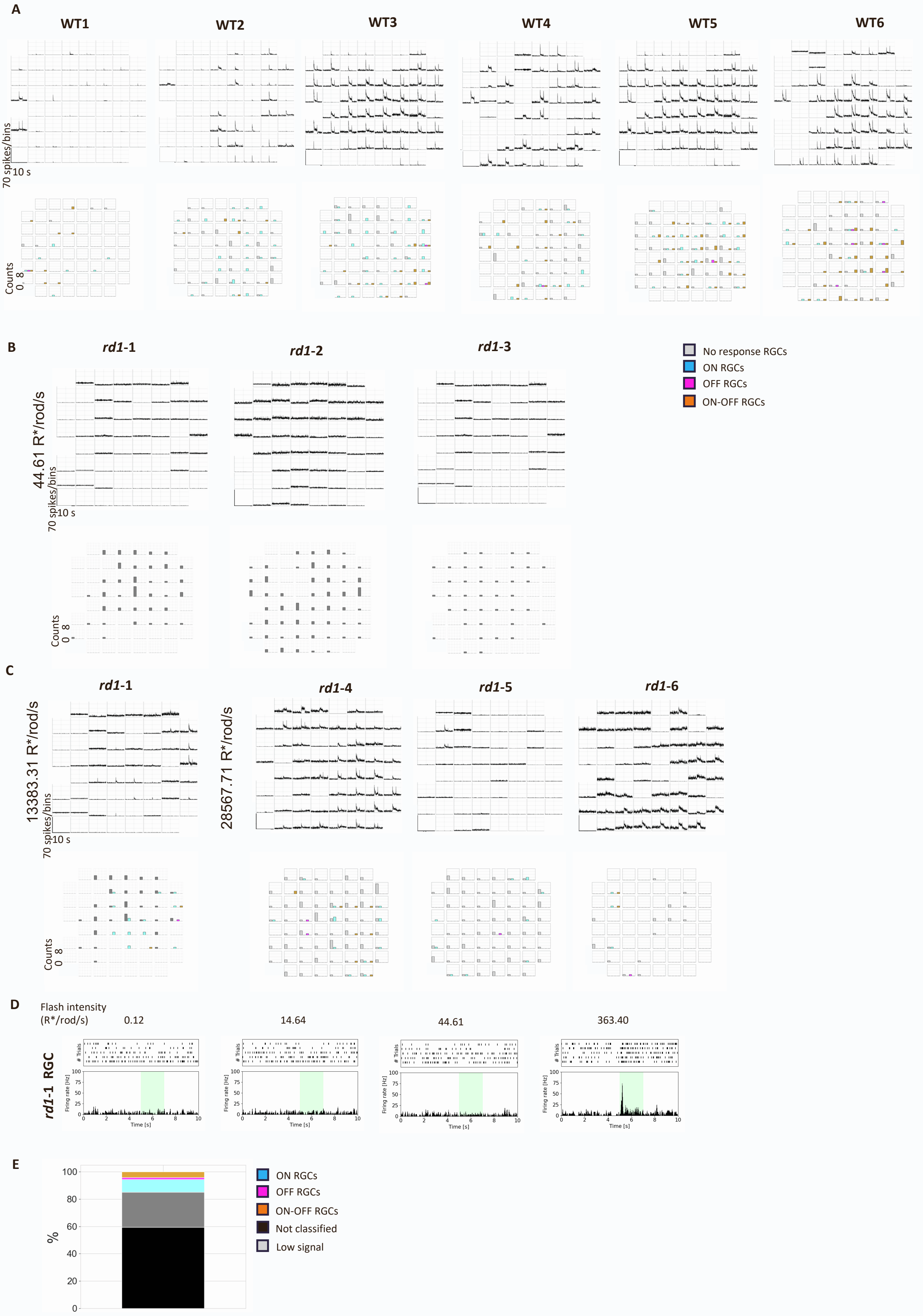

Figure S3. Summary of each WT and *rd1* retina recorded by MEA

(A-C) The time histogram of spikes recorded from each electrode and distribution map illustrating the counts of each RGC type after spike sorting in WT retinas (A:6 retinas, 6 animals), 4-6 weeks *rd1* retinas(B: 3 retinas ,3 animals, C: 4 retinas, 4 animals).

(D) Representative light responses of RGC in 6 week *rd1* retina.

(E) RGC types in 4-6 weeks *rd1* retinas (288 cells from 3 retinas, 3 animals).

Based on the shape of the response to a 2-s flash (28567.71 R*/rod/s), each RGC was classified as ON(27 cells), OFF (4 cells), ON-OFF(12 cells), Low-signal (74 cells), or Not classified (171 cells).

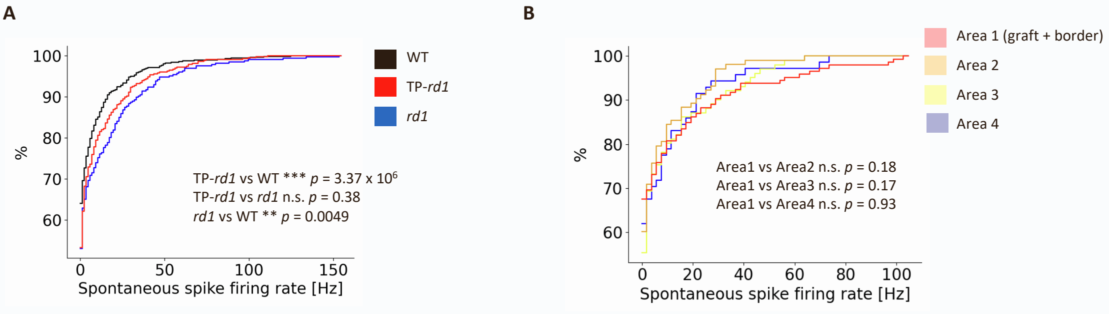

Figure S4. Spontaneous activity in the dark from WT, *rd1* and TP-*rd1* retinas

(A) Cumulative probability of 10-second mean spontaneous spike firing rate under dark from WT(556 RGCs, 6 retinas, 6 animals), the TP-*rd1* (413 RGCs, 6 retinas, 6 animals) and the *rd1* (326 RGCs, 4 retinas, 4 animals) retinas.

(B) Cumulative probability of 10-second mean spontaneous spike firing rate under dark from Area1 (147 RGCs), Area2 (102 RGCs), Area3 (94 RGCs) and Area4 (70 RGCs) in the TP-*rd1* retinas (6 retinas, 6animals).

Table S1. Summary of each TP-*rd1* retina used for the experiment

Sample	Host	Age at analysis (Weeks)	Age at time of transplantation (Weeks)	Post-TP (weeks)	graft Cell Line	Graft Age	No. of Electrodes				No.of light-responded RGCs / ALL RGCs after spike sorting			
							Area1	Area2	Area3	Area4	Area1	Area2	Area3	Area4
TP1	*rd1*	19	10	9	*Islet1* $^{-/-}$	dd13	15	15	14	8	0/8	2/5	0/1	0/1
TP2	*rd1*	25	19	6	*Islet1* $^{-/-}$	dd13	15	17	16	8	9/28	7/16	4/18	0/5
TP3	*rd1*	22	14	8	*Islet1* $^{-/-}$	dd13	13	17	13	12	12/28	4/27	1/15	2/24
TP4	*rd1*	22	14	8	*Islet1* $^{-/-}$	dd13	20	15	13	9	12/32	8/20	2/11	2/11
TP5	*rd1*	19	10	9	*Islet1* $^{-/-}$	dd13	13	13	17	15	12/23	4/17	1/28	0/23
TP6	*rd1*	18	10	8	*Islet1* $^{-/-}$	dd13	20	15	15	9	4/28	2/17	1/21	0/6
TP7*	*rd1*	46	32	14	*Islet1* $^{-/-}$	dd13	-	-	-	-	-	-	-	-

*TP7 was utilized solely for the histological analysis presented in Figure S1.

Table S2. Summary of Mann–Whitney U test in Figure3D

Flash intensity R*/rod/s	p-values	Number of RGCs	
		WT	TP-*rd1*
1.49	1.08×10^{-9}	175	25
4.32	1.76×10^{-8}	224	23
15.32	2.59×10^{-6}	251	62
49.38	0.0047	251	66
95.22	1.29×10^{-5}	279	84

Supplemental procedures

Mouse ES cell line and retinal organoid differentiation

On differentiation day (DD) 0, mouse ES cells were plated at 5,000 cells/well in 96-well plates (Thermo, 174925) to form aggregates in differentiation medium (Glasgow minimum essential medium [GIBCO, 11710035], 5 % KSR [GIBCO, 10828-028], 0.1 mM nonessential amino acids [GIBCO, 11140-050], 1 mM pyruvate [Sigma, S-8636], and 0.1 mM 2-mercaptoethanol [Wako,137-06862]) + 100 μM AGN193109 (Toronto Research Chemicals, A427000). The aggregates were incubated at 37 °C with 20 % O_2 and % CO_2. On DD1, growth factor-reduced Matrigel (2 %, BD Biosciences, 354230) was added to the differentiation medium. On DD7, aggregates were transferred to the retinal maturation medium (DMEM/F12 with GlutaMAX [GIBCO, 10565], 1 % N2 supplement [GIBCO, 17502-048] and 1 % penicillin-streptomycin [GIBCO, 15140-122]) in a 6-cm dish (CORNING, 351007) and incubated at 37 °C with 40 % O_2 and 5 % CO_2. On DD11, all-trans retinoic acid (0.5 μM, Sigma, R2625-100MG) and L-taurine (1 mM, Sigma, T8691) were added to the retinal maturation medium. On DD13, a small piece (approximately 1 × 0.5 mm) containing a characteristic continuous neural epithelial structure was excised from each optic vesicle for transplantation (Assawachananont et al. 2014).

Transplantation of gRO sheets

Injection tips were prepared from disposable micropipettes (Drummond, 1-000-0500) using a micropipette puller (Sutter Instrument, P-97/IVF Puller), and the tip (approximately 500 μm in diameter) was sharpened using a microgrinder (Narishige, EG-400). The injection tip was then fixed in a microelectrode holder (World Precision Instruments, MPH310) on the electrode handle (World Precision Instruments, 2505), which was connected to an extension tube, and the route was filled with HBSS (Gibco, 14170112) using a 1-mL syringe. Then, the syringe was replaced

by a 10-µl micro-syringe (Hamilton, 1701LT), and the gRO sheets were loaded in the injection tip by aspiration with approximately 2 µL 6x Viscoat, hyaluronate and chondroitin sulfate sodium (Alcon). Mice were anesthetized with a mixture of medetomidine hydrochloride (0.75 mg/kg body weight; Nippon Zenyaku Kogyo Co., Ltd., Domitor), midazolam (4 mg/kg body weight; Maruishi Pharmaceutical, Dormicum) and butorphanol tartrate (5 mg/kg body weight; Meiji Animal Health, Vetorphal). The eyes were dilated with 0.4 % tropicamide (Rohto Nitten). Two small scleral punctures were made at the peripheral retina with a 30 G needle, one for graft injection and the other for reducing intraocular pressure, and the gRO sheets were gently injected subretinally into the eyes of *rd1* or *L7*-GFP:*rd1* mice.

Immunohistochemistry

After MEA recordings, the retina was removed from the MEA, washed with phosphate buffered saline (PBS) and fixed with 4% paraformaldehyde (Wako Pure Chemical Industries, 30525-89-4) for 15 min at room temperature (RT). The retina was then incubated in blocking buffer (3 % Triton X-100 and 1 % bovine serum albumin [Sigma, A4503]) for 1 h at RT, and then with the primary antibody against mGluR6 (1:2000) (Leinonen et al., 2020) for 1 week at 4 °C. The retina was washed three times with blocking buffer, followed by incubation with secondary antibody (Alexa Fluor 647 Donkey Anti-Sheep IgG (H+L), Invitrogen, A21448) and DAPI (Invitrogen, D1306) for 3 days at 4 °C.

For immunostaining of the TP-*rd1* retinal sections, mice were sacrificed by cervical dislocation and the eyes were enucleated. The lenses were excised, and the eyes were immediately fixed in 4 % paraformaldehyde for 15 min. The eyes were then washed with PBS and immersed in a 30 % sucrose solution overnight at 4 °C. Subsequently, they were embedded in optimal cutting temperature compound (4583, Sakura Finetek, Tokyo, Japan) and stored at -30 °C. Coronal

cryosections of 14 μm thickness were prepared using a cryostat (Thermo Scienctific CryoStar NX70). The sections were then incubated in blocking buffer (3 % Triton X-100 and 1 % bovine serum albumin [Sigma, A4503]) for 1 h at room temperature (RT), followed by incubation with primary antibodies against cone arrestin (Millipore, AB15282), rhodopsin (sigma, 04886), L/M opsin(Opsin Red/Green, Millipore, AB5405), and S-opsin(OPN1SW, SantaCruz, sc-14363) for 3 days at 4 °C. The retina was washed three times with the blocking buffer, followed by incubation with secondary antibody donkey-anti-Rb-647 (Invitrogen, A31573), donkey-anti-mouse-647 (Invitrogen, A31571), donkey-anti-Goat-647 (Invitrogen, A21447), and DAPI (Invitrogen, D1306) for 1 day at 4 °C.

After the secondary antibody incubation, the retina was washed three times with blocking buffer and subsequently mounted on a glass slide using Vectashield (Vector Laboratories, H-1000). Z-stack Images were acquired with a Leica TCS SP8 confocal microscope and reconstructed in 3D using Imaris Microscopy Image Analysis Software (Oxford Instruments, http://www.bitplane.com/). An overall view of the engrafted whole-mount retina was obtained using a BZ9000 (Keyence).

Spike sorting

The raw data were band-pass filtered to extract components between 100 and 3,000 Hz. The maximum negative value below the threshold defined by the following formula was detected as the spike peak (Quiroga et.al., 2004).

$$threshold = -4 \times median$$

where *x* represents each data point. The first and second differences between data points were calculated from data points between −1 and 2 ms from the peak of the detected spike, and the dimensions were converted to two dimensions using principal component analysis. Clustering

was performed using density-based spatial clustering of applications with noise as a feature (HDBSCAN) of each waveform. Furthermore, using a template matching method (Zhang et al., 2004), we implemented an algorithm to merge clusters and another to rescue spikes from a group of waveforms considered as noise into one of the clusters. To verify the accuracy of spike sorting, we performed autocorrelation analysis on the sorted spike train from each unit and confirmed the presence of a refractory period (± 1 ms).

Light simulation for MEA recordings

In Figure 2, a 2-s flash (95.22 R*/rod/s) was presented under the dark background. In Figure 3, to examine the light intensity-response relationship, the 2-s flash intensity was varied from 0.83 to 95.22 R*/rod/s. In a similar experiment, the *rd1* retina (4–6-week-old) was stimulated by light (24.85 to 28567.71 R*/rod/s). In Figures 4–6, the background light was applied for more than 5 min before the start of the measurements. In Figure 4, a 2-s flash (18794.68 to 43944.81 R*/rod/s) was applied under the photopic light background (15441.33 R*/rod/s). In Figure 5 and 6, a 10-ms square wave stimulus was flickered at 5, 15, 20, and 25 Hz. The light intensities used were: 15.32 R*/rod/s flicker stimulus under the dark background condition, 95.23 R*/rod/s flicker stimulus under the dark background condition, and 43944.81 R*/rod/s flicker stimulus under the 1189.59 R*/rod/s background light condition. In the actual experiment, after completing the stimulation protocol under the dark background, the samples were given a 5-min adaptation period to the light background, followed by the administration of the stimulation protocol under the light background. A 2-s flash (0.12 to 2856.71 R*/rod/s) under the dark background is shown in Figure S3.

Supplemental references

Assawachananont, J., Mandai, M., Okamoto, S., Yamada, C., Eiraku, M., Yonemura, S., Sasai, Y., and Takahashi, M. (2014). Transplantation of embryonic and induced pluripotent stem cell derived 3D retinal sheets into retinal degenerative mice. Stem Cell Reports. *2*, 662–674._ https://doi.org/10.1016/j.stemcr.2014.03.011.

Leinonen, H., Pham, N. C., Boyd, T., Santoso, J., Palczewski, K., & Vinberg, F. (2020). Homeostatic plasticity in the retina is associated with maintenance of night vision during retinal degenerative disease. Elife, 9, e59422. https://doi.org/10.7554/eLife.59422

Quiroga, R. Q., Nadasdy, Z., and Ben-Shaul, Y. (2004). Unsupervised spike detection and sorting with wavelets and superparamagnetic clustering. Neural Comput, 16, 1661-—1687. https://doi.org/10.1162/089976604774201631.

Zhang, P. M., Wu, J.- Y., Zhou, Y., Liang, P.- J., and Yuan, J.- Q. (2004). Spike sorting based on automatic template reconstruction with a partial solution to the overlapping problem. J Neurosci Methods, 135, 55-—65. https://doi.org/10.1016/j.jneumeth.2003.12.001.
